# Substrate preference of protein kinase B isoforms can vary depending on the cell line

**DOI:** 10.1371/journal.pone.0298322

**Published:** 2024-03-19

**Authors:** Miguel S. Palma, Samantha R. Perez, Aida Husain, Deepali Bhandari

**Affiliations:** Department of Chemistry and Biochemistry, California State University Long Beach, Long Beach, California, United States of America; NCI: National Cancer Institute, UNITED STATES

## Abstract

Many proteins in higher eukaryotes, especially those with crucial functions, have multiple isoforms with redundant roles providing protection against potential functional deficiencies in one isoform. However, these isoforms can also have some unique roles. Protein kinase B, also known as Akt, is one such protein that has three isoforms encoded on different genes. Due to high sequence similarity and the general lack of specific reagents, most studies on Akt generalize their findings and do not distinguish between the isoforms. Using an established chemical genetic strategy and a set of known Akt substrates, this work explores substrate specificity of Akt isoforms under steady state conditions in two commonly used cell lines. This strategy can be applied to study any Akt isoform-specific substrates of interest in any cell line of choice as long as the cell line can be transfected.

## Introduction

Protein kinase B (PKB), a serine/threonine protein kinase, also known as Akt, is a major signaling hub in mammalian cells. It has over ~100 known substrates and is involved in the regulation of major cellular functions including proliferation, migration, survival, and metabolism [1]. The canonical model of Akt activation involves generation of phosphatidylinositol 3,4,5 triphosphate (PIP3) by PI3 kinase on the inner leaflet of the plasma membrane in response to growth factor receptor stimulation [2]. Akt is recruited to the plasma membrane upon binding to PIP3 via its N-terminal pleckstrin homology (PH) domain [3]. At the plasma membrane, Akt is phosphorylated on two sites–a threonine residue in the catalytic domain by the phosphoinositide-dependent kinase 1 [4,5] and a serine residue in the C-terminal hydrophobic domain primarily by the mammalian target of rapamycin complex 2 [6]. Phosphorylation on these two residues maximally activates Akt which in turn phosphorylates and regulates activity/function of its downstream substrates [2,7].

There are three isoforms of Akt encoded in the mammalian genome–Akt1 (PKBα), Akt2 (PKBβ), and Akt3 (PKBγ). Each of the three isoforms is encoded on a separate gene–Akt1 on chromosome 14q32, Akt2 on 19q13, and Akt3 on 1q44 [8–10]. While Akt1 and Akt2 are ubiquitously expressed, expression of Akt3 is limited to specific tissues and is especially enriched in the brain [11]. Akt1 is the most well studied member of the family and is still often simply referred to as Akt in literature. Despite their overall high sequence similarity (~80%) and conserved tertiary structure [12], non-redundant functions of Akt isoforms have been reported. Based on knock-out mouse models as well as *in vitro* studies, Akt1 has been shown to be involved in regulation of cell survival [13,14], Akt2 in regulation of glucose homeostasis (insulin signaling) [15,16], and Akt3 in brain development [17,18]. Comparison of single and double knock-out studies have revealed the ability of Akt isoforms to compensate for each other to some extent [19–22]. Interestingly, in some cases, opposing functions of the isoforms have been reported, especially in breast cancer studies where Akt1 has been shown to suppress and Akt2 has been shown to promote metastasis [23–26]. Given its involvement in a diverse repertoire of vital cellular functions, Akt is of therapeutic interest in several diseases [27]. Therefore, it is important to be able to study substrate specificity and/or overlap among the Akt isoforms.

Isoform-specific knockout and knockdown strategies provide valuable insight into their individual functions. However, generation of a knockout cell line is time consuming and siRNA-mediated knock-down can be sub-optimal. An alternative approach is a previously described chemical genetics strategy that utilizes expression of Akt isoform constructs containing a tryptophan to alanine mutation at a conserved position in the PH domain [28,29]. The W-to-A substitution makes Akt refractory to an allosteric pan-Akt inhibitor, MK-2206. While the endogenous Akt isoforms are inactivated by MK-2206, the expressed construct of Akt harboring the W-to-A substitution remains functional; thus, allowing to study rescue of substrate phosphorylation by that isoform. Here, we used this strategy to look at isoform contribution to phosphorylation of well-known substrates of Akt in two widely used cell lines (HEK293T and HeLa) under steady state conditions. Our results reveal that the substrate preference for Akt isoforms can be cell-line specific. These findings also suggest that this approach can be used to test/confirm isoform-specificity of Akt substrates in any transfectable cell line of choice.

## Materials and methods

### Reagents and antibodies

All reagents and chemicals were of analytical grade. Akt allosteric inhibitor MK-2206 (#11593) was bought from Cayman Chemical Company. Antibodies against Akt1 (#sc-5298), Akt2 (#sc-5270), Akt3 (#sc-134254), Glyceraldehyde 3-phosphate dehydrogenase (GAPDH; #sc-47724), Haemagglutinin (HA; #sc7392) and Translocase of the mitochondrial outer membrane 40kDa (TOM40; #sc-365467) were from Santa Cruz Biotechnology. Antibodies against, Pan-Akt (#4691), Pan-phospho-T308-Akt (pT308-Akt; #13038), Pan-phospho-S473-Akt (pS473-Akt; #4060), Akt substrate of 160kDa (AS160; #2670), phospho-AS160-Thr642 (pAS160; #8881), Forkhead box class O3a (FoxO3a; #12829), phospho-FoxO3a-Ser253 (pFoxO3a; #13129), Glycogen synthase kinase-3 beta (GSK3β; #12456), phospho-GSK3β-Ser9 (pGSK3β; #5558), proline-rich Akt substrate of 40kDa (PRAS40, #2691), phospho-PRAS40-Thr246 (pPRAS40; #2997), HA (#3724S) and Histone H3 (#4499) were from Cell Signaling Technology. IRDye 680RD and IRDye 800CW secondary antibodies for immunoblotting were from Li-COR Biosciences (#925–68070, #925–68071, #925–32210, #925–32211).

### Plasmid constructs

pcDNA3.1-HA-Akt1 construct was a gift from Dr. Jaewhan Song (Addgene plasmid #78778). The HA tag at the 5’ end was cloned between *Kpn*I and *EcoR*I sites followed by human Akt1 cDNA between *EcoR*I and *Xho*I [30]. Akt2 and Akt3 cDNAs were amplified via polymerase chain reaction (PCR) using Addgene plasmids #86593 and #9017, respectively, as templates. Plasmid #86593 (pEGFP-Akt2) was a gift from Drs. Thomas Leonard and Ivan Yudushkin [31]. Plasmid #9017 (pcDNA3 Myr-HA-Akt3) was a gift from Dr. William Sellers. The restriction digested PCR products of Akt2 and Akt3 cDNAs were cloned into the same backbone as pcDNA3.1-HA-Akt1 between the *EcoR*I and *Xho*I sites. The constructs HA-Akt1 W80A, HA-Akt2 W80A and HA-Akt3 W79A were generated using the QuikChange Lightning Site-Directed Mutagenesis Kit (Agilent). The mutagenizing primers were generated using the Agilent QuikChange Primer Design tool. All primers were ordered from Integrated DNA Technologies. The mutations and the integrity of the rest of the cDNA were confirmed by sequencing at Genewiz.

### Cell culture and transfection

HEK293T and HeLa cells (American Type Culture Collection) were cultured in Dulbecco’s Modified Eagle Medium (Gibco #11965092) supplemented with 10% Fetal Bovine Serum (Cytiva Hyclone #SH30071.03) and Penicillin, Streptomycin, and Glutamine (Corning #30009CI). Cells were maintained in a humidified atmosphere containing 5% CO_2_ at 37°C. Transfections were performed using TransIT-LT1 transfection reagent (Mirus Bio #MIR2300) according to the manufacturer’s protocol.

### Cell lysis and immunoblotting

Cells were lysed in cold lysis buffer (25mM HEPES pH 7.4, 1%TritonX100, 150mM NaCl, 1mM MgCl_2_, protease and phosphatase inhibitors) and the lysates cleared by centrifugation at 15,000 rpm for 10 minutes at 4°C in a refrigerated microcentrifuge (Eppendorf #5425R). Laemmli buffer was added at 1X final concentration to the cleared lysates followed by heating at 95°C for 5 minutes. Samples were analyzed by immunoblotting following the standard protocol as previously described [32]. Briefly, samples were separated using sodium dodecyl sulfate-polyacrylamide gel electrophoresis under reducing conditions and electrotransferred to Immobilon^TM^ FL PVDF membranes (EMD Millipore #IPFL00010) using the Mini Trans-Blot Electrophoretic Transfer Cell Transblot system (Biorad #1703930). After the transfer was complete, membranes were blocked with phosphate buffered saline supplemented with 5% nonfat milk and then incubated sequentially with primary and secondary antibodies. Infrared imaging with two-color detection and quantification of blots were performed according to the manufacturer’s protocols using Odyssey Fc imaging system (Li-COR Biosciences).

### *In vitro* kinase assays

Lysates of cells transfected with HA-Akt constructs were immunoprecipitated with the mouse monoclonal anti-HA antibody (#sc7392). The immunoprecipitates were used to perform *in vitro* kinase assays on purified recombinant PRAS40 (residues 182–256) purchased from Novus Biologicals (catalog # NBP2-57165PEP). PRAS40 protein (0.5 μg) was incubated with HA-Akt immunoprecipitates in 25 μL of kinase buffer (50 mM HEPES pH 7.4, 1 mM DTT, 5 mM MgCl_2_, 5 mM MnCl_2_, 5 mM ATP, Protease and Phosphatase Inhibitors) at 30°C for 15, 30, 60 or 90 minutes. Reactions set up in the buffer without ATP were included as controls. The reactions were stopped by adding the Laemmli buffer at 1X final concentration and the samples were analyzed by immunoblotting.

### Cell fractionation

HEK293T or HeLa cells either untransfected or transfected with the HA-Akt isoform constructs were fractionated into nuclear, cytosolic and mitochondrial fractions using the cell fractionation kit from Abcam (catalog #ab109719) following the manufacturer’s protocol. The fractions were subsequently analyzed by immunoblotting. Histone H3, GAPDH and TOM40 were blotted as the nuclear, cytosolic and mitochondrial controls, respectively.

### Statistical analysis

The band intensities were quantified using ImageStudio Lite Quantitation Software (LI-COR Biosciences). The phospho-protein band intensities were normalized to total protein levels which were then compared among the DMSO and MK-2206 treated sets expressing either WT or W80/79A Akt isoform. All graphical data were prepared, and statistical analyses were performed using the GraphPad Prism software.

## Results and discussion

### HEK293T cells express all three Akt isoforms which are optimally inhibited by the allosteric inhibitor MK-2206

**Fig 1:**
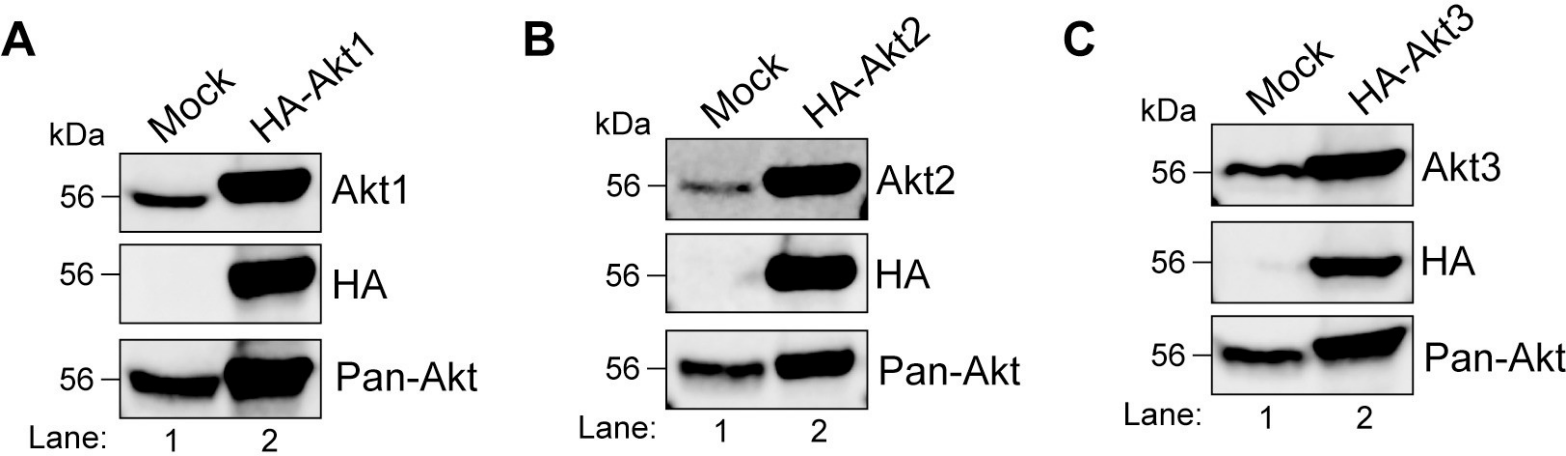
HEK293T express all three isoforms of Akt. HEK293T cells were either mock-transfected or transfected with HA-Akt1 **(A)**, HA-Akt2 **(B)**, or HA-Akt3 **(C)** for 24h prior to lysis. Cell lysates were analyzed by immunoblotting. The isoform specific antibodies and the ​pan-Akt antibody detected both endogenous and transiently overexpressed HA-tagged isoforms. The HA antibody confirmed expression of the Akt constructs. The predicted molecular masses of the proteins are indicated. kDa = kilodalton.

**Fig 2:**
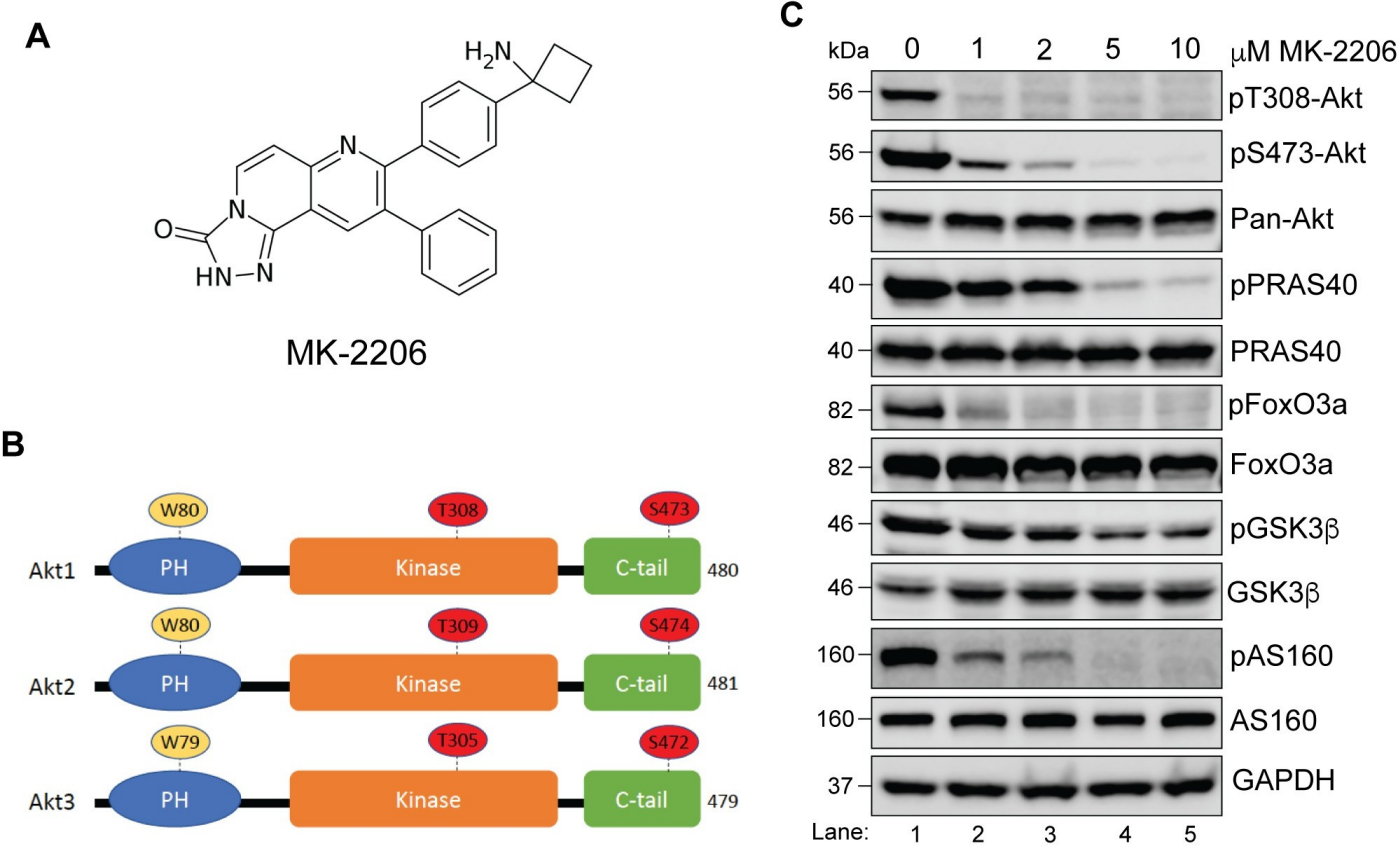
Dose response of MK-2206 based inhibition of Akt in HEK293T cells. **A.** Structure of MK-2206. **B.** Schematic of the domain architecture of Akt isoforms. The N-terminal PH domain, central kinase domain and the C-terminal hydrophobic tail are shown. The residues important for MK-2206 binding (W80 in Akt1 and Akt2, W79 in Akt3) and for activation of the kinase (T308 and S473 in Akt1, T309 and S474 in Akt2 and T305 and S472 in Akt3) are indicated. The domains are not drawn to scale. Total number of residues in the three isoforms is indicated. **C.** HEK293T cells were treated with the indicated concentrations of MK-2206 for 3h before lysis. Cell lysates were analyzed for pAkt (T308 and S473), pan-Akt, and total and phosphoproteins for PRAS40, GSK3β, AS160, and FoxO3a by immunoblotting. GAPDH was blotted as a loading control.

To carry out substrate specificity analysis of Akt isoforms under steady state conditions, we first used **h**uman **e**mbryonic **k**idney (HEK)293T cells. These cells express all three Akt isoforms endogenously as determined by immunoblotting (Fig 1A–1C, Lane 1). Lysates of HEK293T cells transfected with HA-Akt1, 2 or 3 constructs were used as positive controls (Fig 1A–1C, Lane 2). We next sought to determine the optimal concentration of MK-2206 (structure shown in Fig 2A) for Akt inhibition under steady state (grown and maintained in 10% serum) conditions. HEK293T cells were treated with 0–10 μM of MK-2206 for 3h followed by immunoblotting to determine the level of Akt activation by using pan antibodies that detect phosphorylation on the active site threonine residue—T308 in Akt1, T309 in Akt2, T305 in Akt3, and serine residue in the C-terminal hydrophobic domain—S473 in Akt1, S474 in Akt2, and S472 in Akt3 (shown in Fig 2B schematic). For simplification, we will use Akt1 numbering T308 and S473 to denote these residues in the rest of the manuscript. In addition to Akt, we also determined phospho-status of the four well-documented Akt substrates—PRAS40, GSK3β, FoxO3a, and AS160. A steady decrease in phosphorylation of Akt substrates was observed upon treatment with increasing concentration of MK-2206 which coincided with the reduction in Akt phosphorylation (Fig 2C). Comparable inhibition was seen with 5μM and 10μM of MK-2206 (Fig 2C, lanes 4 and 5). For this reason, we chose 5μM as the optimal concentration of MK-2206 for Akt inhibition in the subsequent experiments. Notably, among the four substrates, phosphorylation of GSK3β was not as reduced as the others. One plausible reason could be that in addition to Akt, S9 on GSK3β has been shown to be phosphorylated by other kinases such as protein kinase A and protein kinase C [33,34].

**Fig 3:**
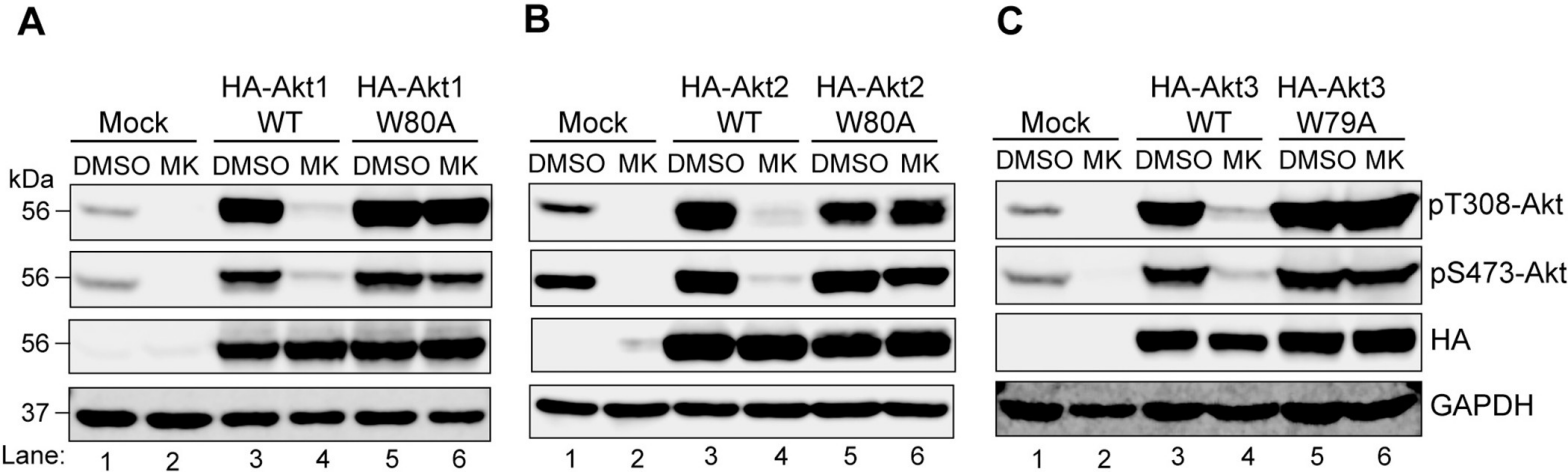
W→A mutation in Akt isoforms resists inactivation by MK-2206 in HEK293T cells cultured under steady state conditions. **A-C.** HEK293T cells were either mock transfected (lanes 1, 2) or transfected with the WT (lanes 3, 4) or W**→**A (lanes 5, 6) constructs of HA-Akt1 **(A)**, HA-Akt2 **(B)**, or HA-Akt3 **(C).** Twenty-four hours post-transfection, cells were treated with DMSO or MK-2206 for 3h, lysed and the cell lysates were analyzed for pAkt (T308 and S473), HA and GAPDH (loading control) by immunoblotting. DMSO = dimethylsulfoxide; MK = MK-2206.

MK-2206 predominantly binds Akt through pi-stacking interactions on a key tryptophan residue within the PH domain (Fig 2B schematic) [35–37]. Therefore, tryptophan to alanine mutation at this position weakens binding of MK-2206 to Akt. We generated N-terminally tagged HA-Akt W79/80A constructs and first confirmed that cells expressing the mutant version of Akt isoforms were indeed resistant to MK-2206 inhibition. As shown in Fig 3A–3C, lysates of mock-transfected cells showed inhibition of Akt activity upon treatment with MK-2206 (compare lanes 1 and 2) as did the cells transfected with wild type (WT) Akt isoforms (compare lanes 3 and 4). However, in cells expressing the W79/80A mutant Akt isoforms (lanes 5 and 6; Fig 3A–3C), MK-2206 failed to reduce T308 and S473 phosphorylation verifying that W79/80A Akt mutants retain activity in the presence of MK-2206.

### Akt1 is the dominant isoform in HEK293T cells under steady state conditions

**Fig 4:**
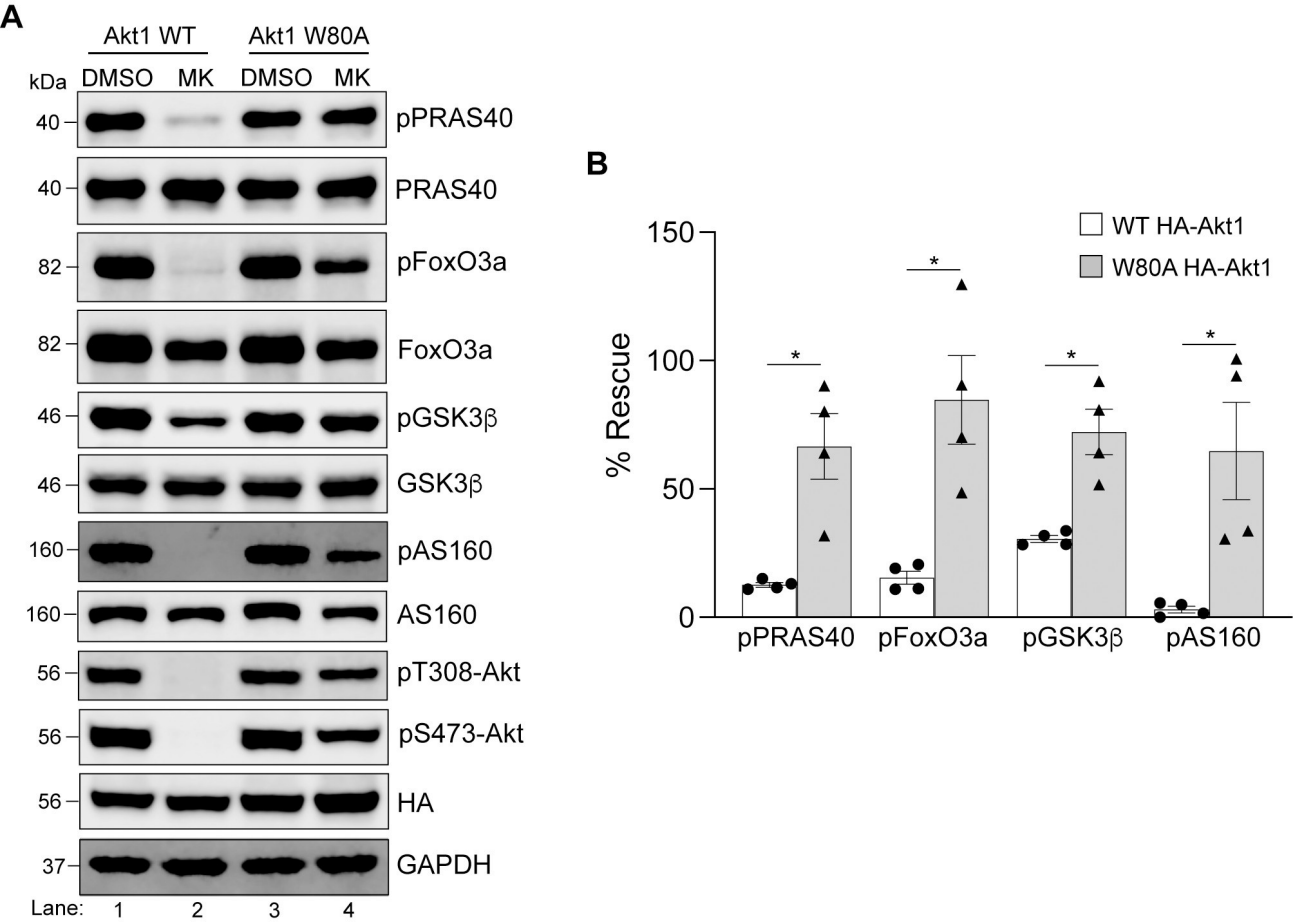
Rescue of substrate phosphorylation in MK-2206 treated HEK293T cells expressing HA-Akt1 W80A. **A.** HEK293T cells were transfected with either HA-Akt1 WT or HA-Akt1 W80A. Twenty-four hours post-transfection, cells were treated with DMSO or MK-2206 for 3h prior to lysis and analyzed by immunoblotting for the indicated proteins. Blots from an experiment representative of four independent experiments are shown. **B.** Phosphorylation of each substrate was quantified and normalized to their total protein expression. The data was graphed comparing phosphorylation of each substrate in the presence of MK-2206 in WT Akt1 expressing cells with that of the W80A Akt1 expressing cells. Error bars represent the standard error of the mean (SEM), n = 4, *p<0.05 (*Mann Whitney test)*.

**Fig 5:**
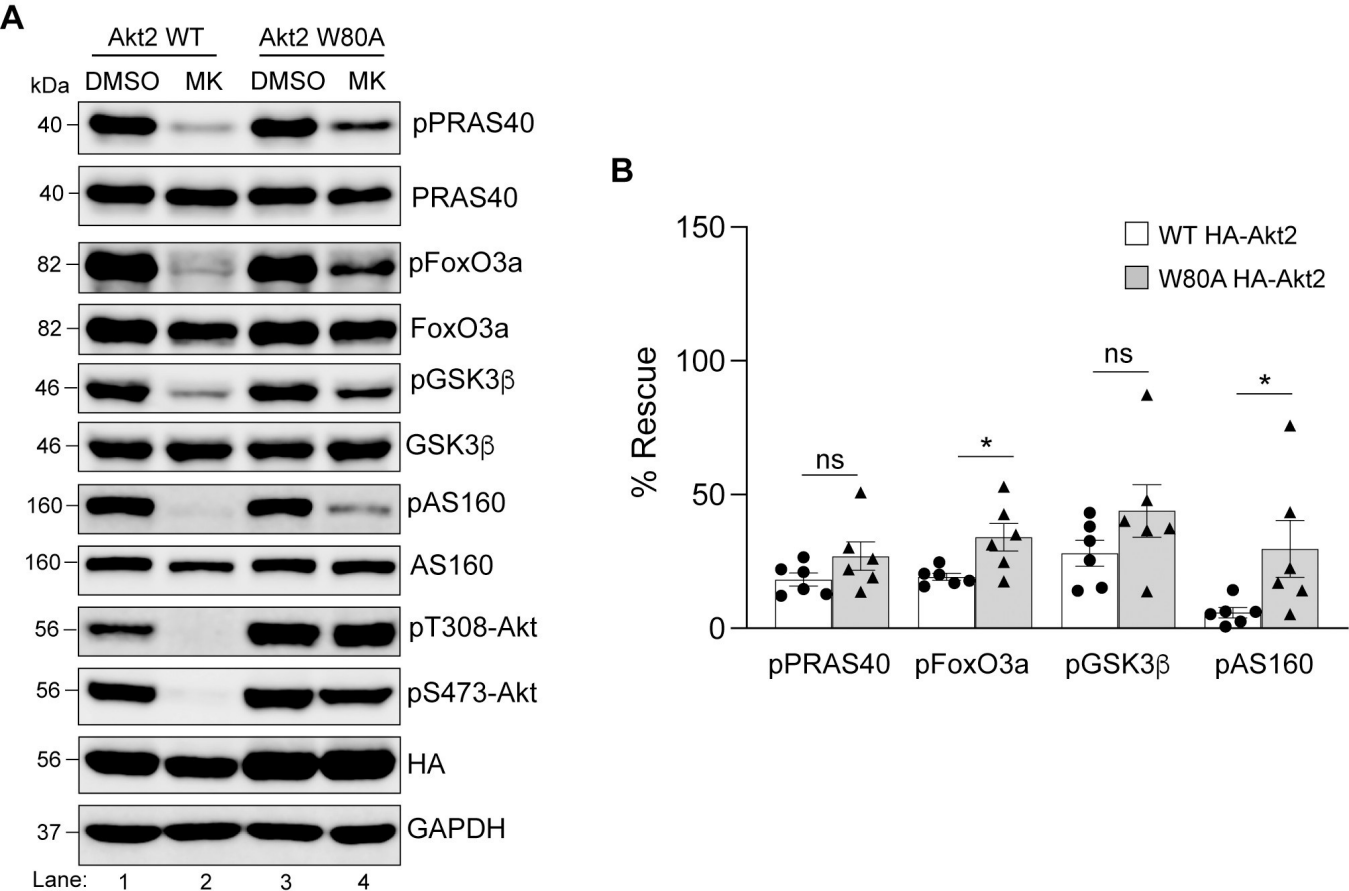
Rescue of substrate phosphorylation in MK-2206 treated HEK293T cells expressing HA-Akt2 W80A. **A.** HEK293T cells were transfected with either HA-Akt2 WT or HA-Akt2 W80A. Twenty-four hours post-transfection, cells were treated with DMSO or MK-2206 for 3h prior to lysis and analyzed by immunoblotting for the indicated proteins. Blots from an experiment representative of six independent experiments are shown. **B.** Phosphorylation of each substrate was quantified and normalized to their total protein level in each category. The data was graphed comparing phosphorylation of each substrate in the presence of MK-2206 in WT Akt2 expressing cells with that of the W80A Akt2 expressing cells. Error bars represent SEM, n = 6, *p<0.05, *ns* = not significant (*Mann Whitney test)*.

**Fig 6:**
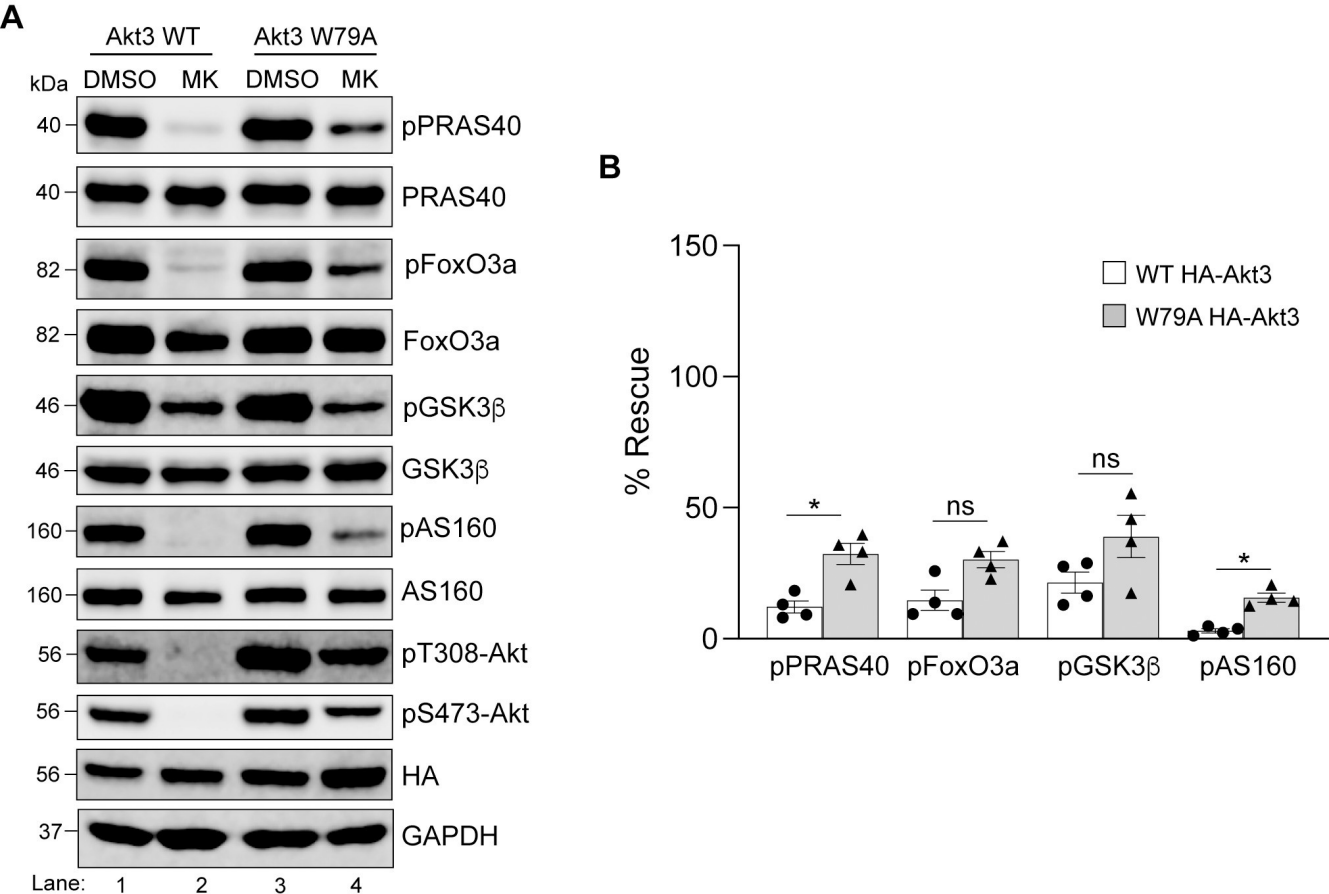
Rescue of substrate phosphorylation in MK-2206 treated HEK293T cells expressing HA-Akt3 W79A. **A.** HEK293T cells were transfected with either HA-Akt3 WT or HA-Akt3 W79A. Twenty-four hours post-transfection, cells were treated with DMSO or MK-2206 for 3h prior to lysis and analyzed by immunoblotting for the indicated proteins. Blots from an experiment representative of four independent experiments are shown. **B.** Phosphorylation of each substrate was quantified and normalized to their total protein level in each category. The data was graphed comparing phosphorylation of each substrate in the presence of MK-2206 in WT Akt3 expressing cells with that of the W79A Akt3 expressing cells. Error bars represent SEM, n = 4, *p<0.05, *ns* = not significant (*Mann Whitney test)*.

Next, we used this approach to determine which isoform(s) rescues phosphorylation of PRAS40, FoxO3a, GSK3β and AS160 in HEK293T cells. All four of these proteins contain validated Akt target sites which reside within the minimum consensus recognition motif R-X-R-X-X-S*/T*ϕ, where X is any amino acid, S/T is the phosphorylated residue and ϕ represents preference for a large hydrophobic residue [1]. Specifically, in this study, we detected phosphorylation of the following residues—T246 on PRAS40, S253 on FoxO3a, S9 on GSK3β, T652 on AS160. HEK293T cells transfected with WT or W→A Akt isoforms were treated with either the solvent dimethylsulfoxide (DMSO) or MK-2206 and the cell lysates were analyzed by immunoblotting for the phosphorylated and total levels of PRAS40, FoxO3a, GSK3β and AS160. As shown in Fig 4A, treatment of cells expressing WT Akt1 with MK-2206 led to a noticeable decrease in phosphorylation of all four substrates (compare lanes 1 and 2) whereas the cells expressing its W80A counterpart showed phosphorylation of each substrate in the absence or presence of MK-2206 (compare lanes 3 and 4). When the phospho-levels were normalized to the total protein for each substrate and plotted as % rescue compared to the DMSO controls, W80A Akt1 showed a significant rescue of each (Fig 4B). We performed similar experiments with Akt2 and Akt3 (Figs 5A and 6A) and observed that cells expressing W→A versions of these two isoforms were not as effective in restoring phosphorylation of the four tested substrates as Akt1 (Fig 4). Even though the analysis (Figs 5B and 6B) showed a significant difference between phosphorylation of some substrates in cells expressing WT *vs* W→A counterparts of Akt2 and Akt3 in the presence of MK-2206 (FoxO3a, AS160 for Akt2; and PRAS40, AS160 for Akt3), the level of phosphorylation recovery was much lower in each case compared to that observed with Akt1 (Fig 4B). Together, these results suggest that Akt1 is the dominant isoform in HEK293T cells for these four substrates under basal conditions.

### HeLa cells only express Akt1 and 2 and both isoforms show redundance in phosphorylation of the tested substrates under steady state conditions

**Fig 7:**
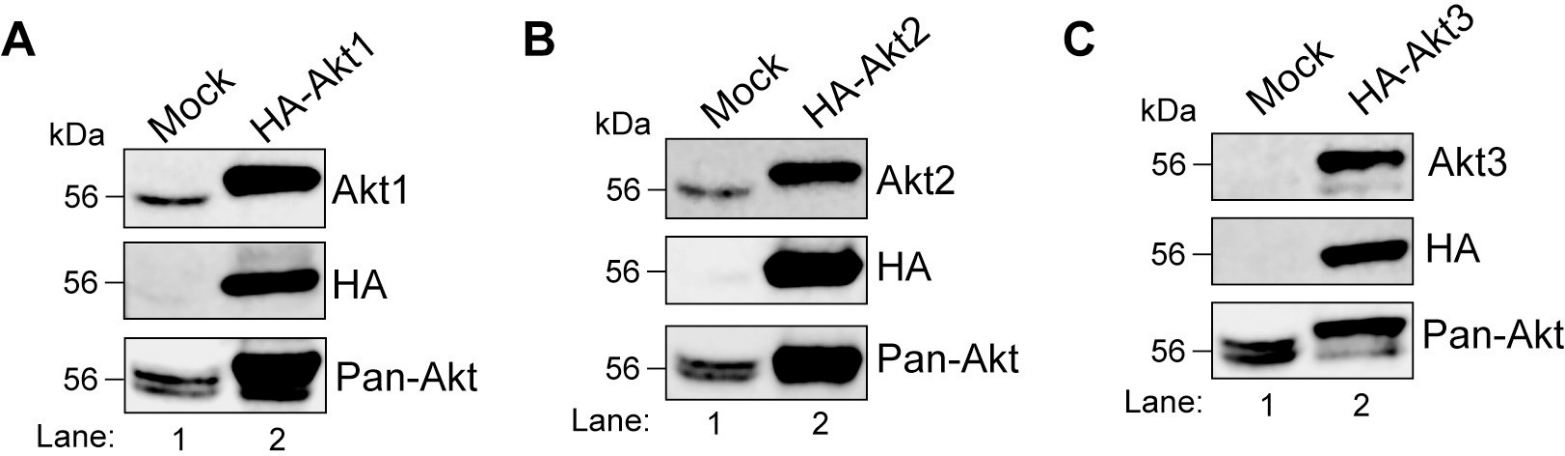
HeLa express two isoforms of Akt. HeLa cells were either mock-transfected or transfected with HA-Akt1 **(A)**, HA-Akt2 **(B)**, or HA-Akt3 **(C)** for 24h prior to lysis. Cell lysates were analyzed by immunoblotting. The isoform specific antibodies and the pan-Akt antibody detected both endogenous and transiently overexpressed HA-tagged isoform. The HA antibody confirmed expression of the Akt constructs.

**Fig 8:**
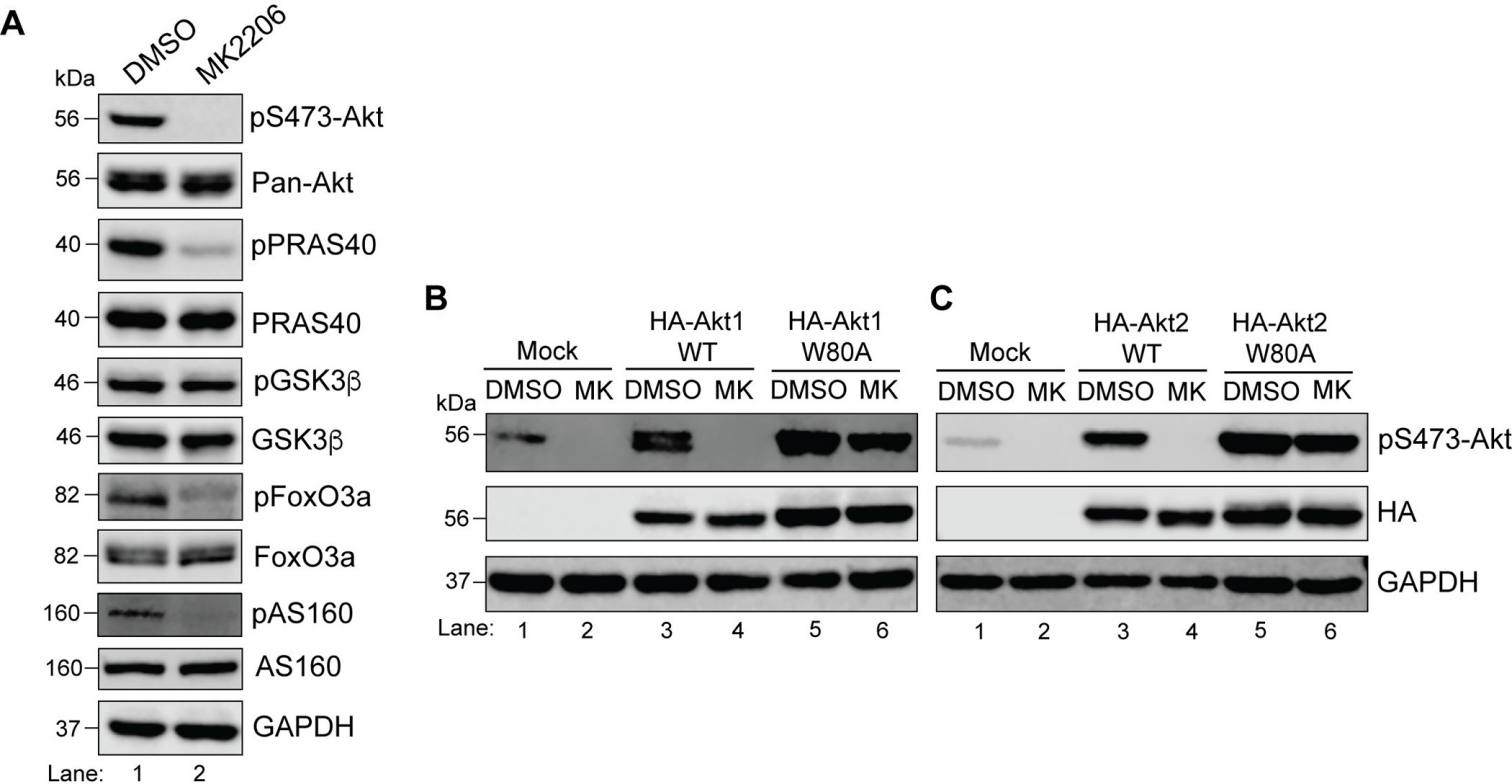
W→A mutation in both isoforms resists inactivation by MK-2206 in HeLa cells cultured under steady state conditions. **A.** HeLa cells were treated with 5μM MK-2206 for 3h before lysis. Cell lysates were analyzed for pAkt (S473), pan-Akt, and total and phosphoproteins for PRAS40, GSK3β, AS160, and FoxO3a by immunoblotting. GAPDH was blotted as a loading control. **B and C.** HeLa cells were either mock transfected (lanes 1, 2) or transfected with the WT (lanes 3, 4) or W**→**A (lanes 5, 6) constructs of HA-Akt1 **(B)**, HA-Akt2 **(C)**. Twenty-four hours post-transfection, cells were treated with DMSO or MK-2206 for 3h, lysed and the cell lysates were analyzed for pAkt (S473), HA and GAPDH (loading control) by immunoblotting.

Next, we used HeLa (cervical cancer cell line originally isolated from Ms. **He**nrietta **La**cks) for a similar analysis as it is another commonly used cell line to study cellular signaling. As with HEK293T cells, we first wanted to determine if HeLa express all three Akt isoforms. Based on the gene expression information extracted from a freely accessible database called DepMap Portal, HeLa only express Akt isoforms 1 and 2. We confirmed this at the protein level by immunoblotting lysates of HeLa cells which were either mock transfected or transfected with HA-Akt constructs. As shown in Fig 7A–7C (lane 1), we could only detect Akt1 and Akt2 in mock transfected HeLa cells as compared to the lysates expressing the recombinant Akt isoforms (lane 2). Next, we determined if treatment with 5μM MK-2206 for 3h was sufficient for Akt inhibition in HeLa cells. As shown in Fig 8A, both Akt phosphorylation on T308 and S473, as well as phosphorylation of PRAS40, FoxO3a, and AS160 were noticeably reduced. However, phosphorylation of GSK3β was not affected. As mentioned earlier, S9 on GSK3β is also phosphorylated by other kinases [33,34]. It is possible that in HeLa, GSK3β is not the main target of Akt. Therefore, we decided to omit GSK3β from our subsequent experiments on HeLa cells.

**Fig 9:**
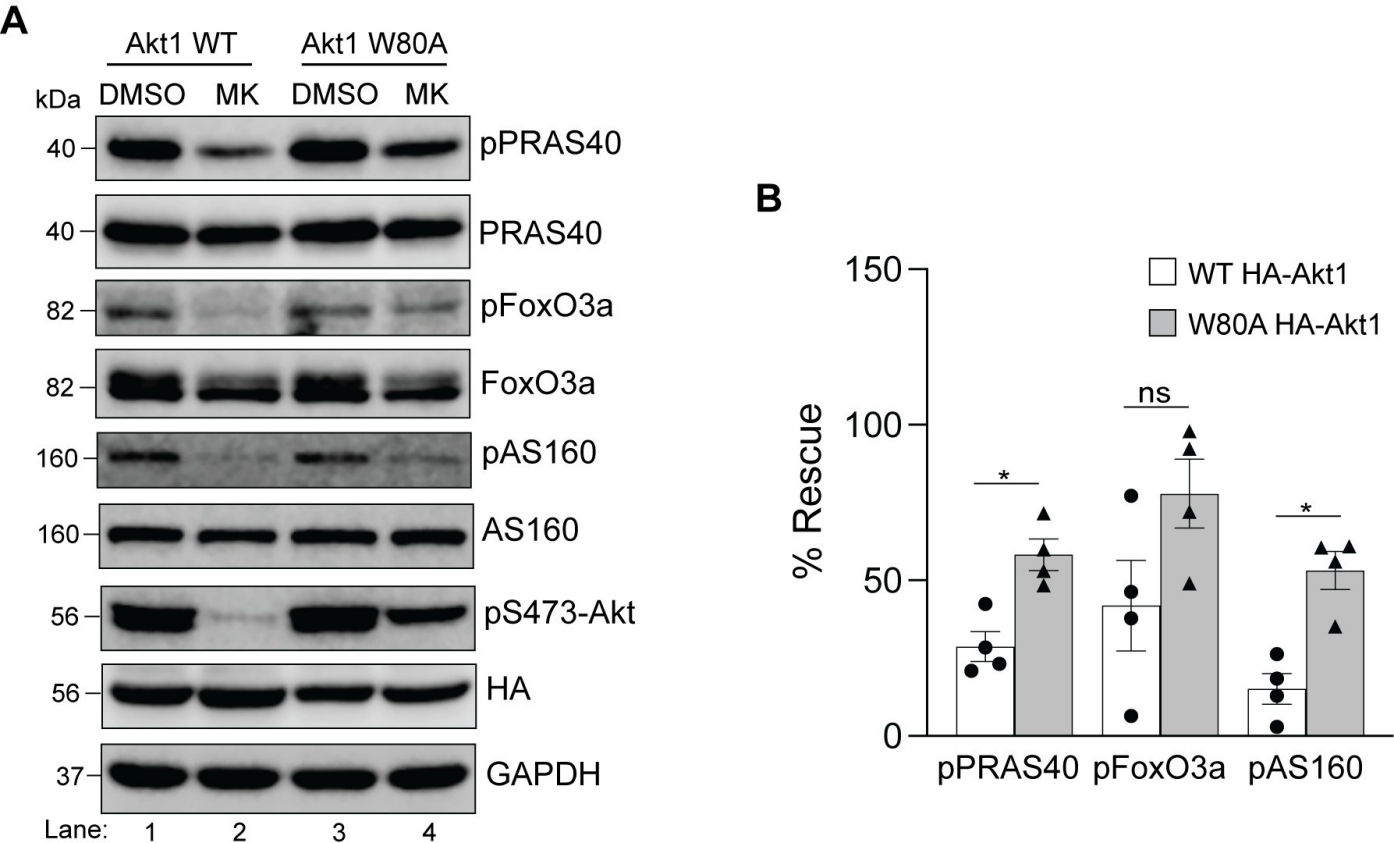
Rescue of substrate phosphorylation in MK-2206 treated HeLa cells expressing HA-Akt1 W80A. **A.** HeLa cells were transfected with either HA-Akt1 WT or HA-Akt1 W80A. Twenty-four hours post-transfection, cells were treated with DMSO or MK-2206 for 3h prior to lysis and analyzed by immunoblotting for the indicated proteins. Blots from an experiment representative of four independent experiments are shown. **B.** Phosphorylation of each substrate was quantified and normalized to their total protein level in each category. The data was graphed comparing phosphorylation of each substrate in the presence of MK-2206 in WT Akt1 expressing cells with that of the W80A Akt1 expressing cells. Error bars represent SEM, n = 4, *p<0.05, *ns* = not significant (*Mann Whitney test)*.

**Fig 10:**
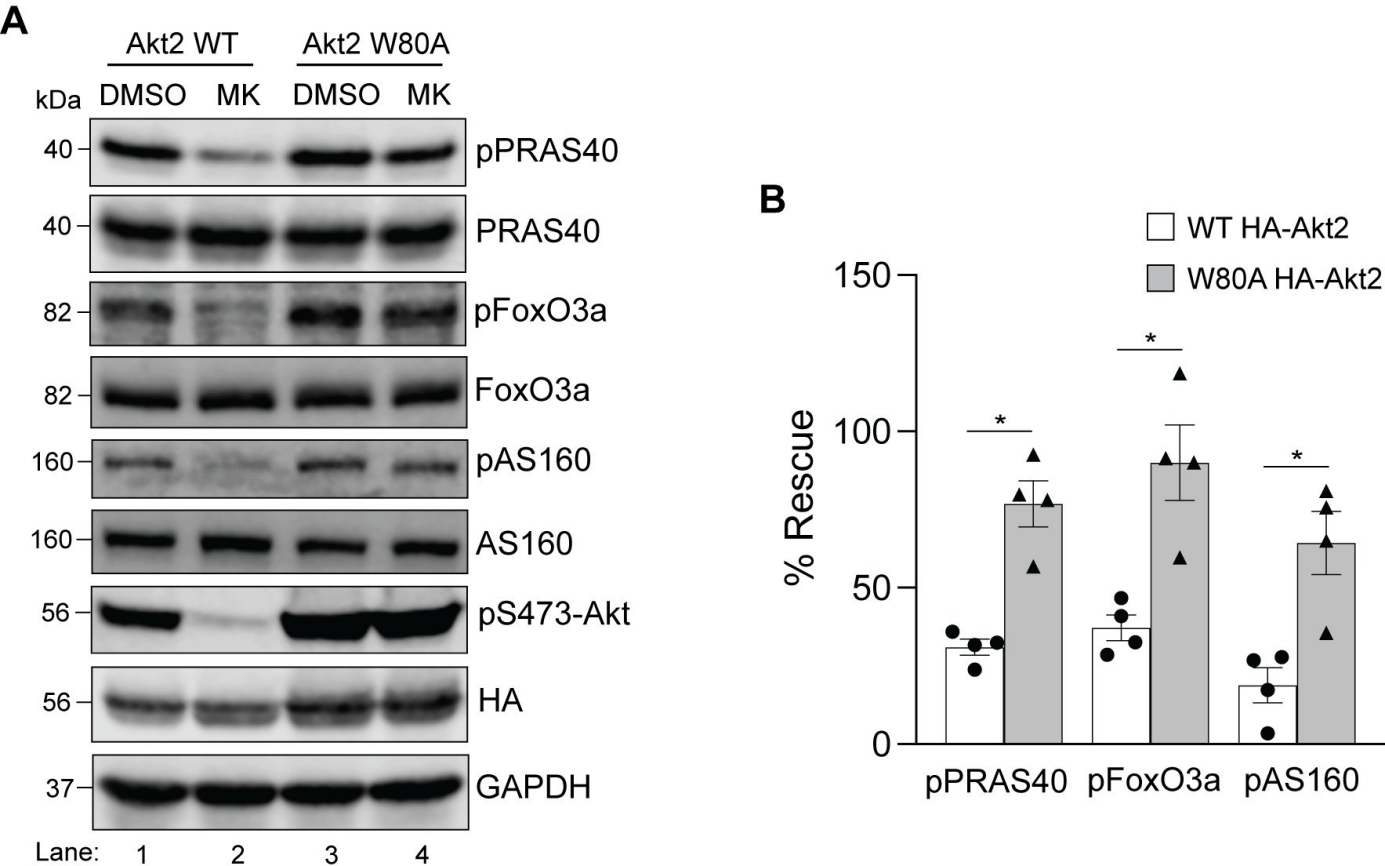
Rescue of substrate phosphorylation in MK-2206 treated HeLa cells expressing HA-Akt2 W80A. **A.** HeLa cells were transfected with either HA-Akt2 WT or HA-Akt2 W80A. Twenty-four hours post-transfection, cells were treated with DMSO or MK-2206 for 3h prior to lysis and analyzed by immunoblotting for the indicated proteins. Blots from an experiment representative of four independent experiments are shown. **B.** Phosphorylation of each substrate was quantified and normalized to their total protein level in each category. The data was graphed comparing phosphorylation of each substrate in the presence of MK-2206 in WT Akt2 expressing cells with that of the W80A Akt2 expressing cells. Error bars represent SEM, n = 4, *p<0.05 (*Mann Whitney test)*.

Using a similar approach as for HEK293T (Figs 4–6), we next assessed whether phosphorylation of PRAS40, FoxO3a, and AS160 was rescued by expression of Akt1 and/or Akt2 in HeLa. Like HEK293T cells, the W80A isoforms in HeLa cells remained active in the presence of MK-2206 (Fig 8B). We subsequently analyzed the phospho-status of the three downstream substrates in HeLa cells expressing either the WT or the W80A constructs of HA-Akt1 (Fig 9) or HA-Akt2 (Fig 10). In contrast to HEK293T cells, both isoforms showed comparable rescue of the three substrates in HeLa cells. Upon quantification, Akt2 W80A appeared to restore phosphorylation of all three substrates better than Akt1 W80A (Figs 9B and 10B), however unlike HEK293T cells, HeLa did not show a clear dominance of one isoform. Together, these results suggest HeLa cells show redundance in the substrate preference of Akt1 and Akt2 for the tested substrates under steady state conditions.

**Fig 11:**
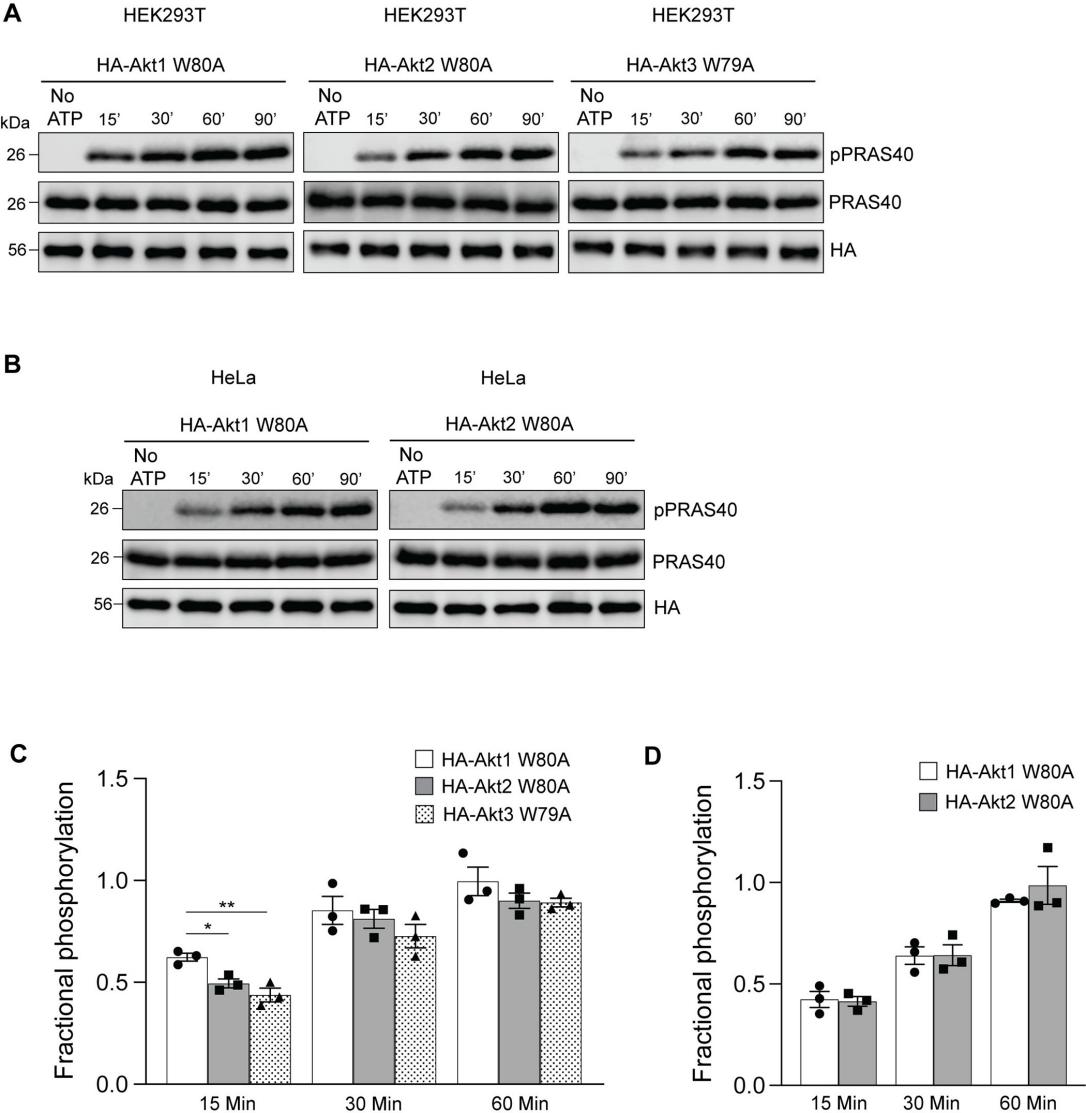
*In vitro* phosphorylation of recombinant, purified PRAS40 by the W→A Akt isoforms. **A and B.** Lysates of HEK293T (A) or HeLa (B) cells transfected with the indicated Akt constructs were immunoprecipitated with the mouse monoclonal anti-HA antibody overnight. The immunoprecipitates were incubated with 0.5 μg of purified recombinant PRAS40 C-terminal fragment in the kinase assay buffer at 30°C for the indicated timepoints. A parallel reaction was set up in the kinase buffer without ATP and incubated for 90 minutes. The reactions were analyzed by immunoblotting for pPRAS40, PRAS40 and HA. **C and D.** The phospho-PRAS40 band was quantified and normalized to the corresponding total PRAS40 protein band in that reaction. The 90-minute timepoint was set at 1 and the fractional phosphorylation at the earlier timepoints was calculated and graphed. An ordinary one-way analysis of variance was performed to determine the statistical significance between the differences of means. Error bars represent SEM, n = 3, *p<0.05, **p<0.01.

One plausible reason for the differences observed in HEK293T and HeLa could be distinct recognition and phosphorylation of the substrates by Akt isoforms in the two cell lines. To test this, we determined whether the W→A Akt isoforms expressed and immunoprecipitated from HEK293T and HeLa cell lines phosphorylated the recombinant carboxyl-terminal (C-terminal) fragment of PRAS40 protein (residues 182–256) in an *in vitro assay*. We performed time-course kinase assays incubating the substrate with HA-Akt immunoprecipitates for 15, 30, 60 and 90 minutes followed by detection of phosphorylation of PRAS40 on residue T246. As shown in Fig 11, all three isoforms of HA-Akt W79/80A in HEK293T (Fig 11A) and both HA-Akt1 W80A and HA-Akt2 W80A expressed in HeLa cells (Fig 11B) phosphorylated PRAS40 starting at the earliest time-point tested (15 min). The reactions appeared to reach maximum at 60–90 minutes. Qualitatively, these results suggested that all the exogenously expressed W→A Akt isoforms are functional and that at least for PRAS40, they are all able to recognize and phosphorylate their target site in an *in vitro* setting. However, when we quantified the fractional phosphorylation at the earlier time-points compared to that at 90 minutes, HA-Akt1 W80A in HEK293T cells showed a significantly higher level of phosphorylation at 15 minutes compared to the other two isoforms (Fig 11C). In contrast, there was no statistically significant difference observed in PRAS40 phosphorylation by the Akt1 and Akt2 isoforms at any time-point in HeLa cells (Fig 11D). These results suggested that Akt1 may be more active and/or bind to PRAS40 better than the other two isoforms in HEK293T cells, contributing to its dominance in this cell line.

**Fig 12:**
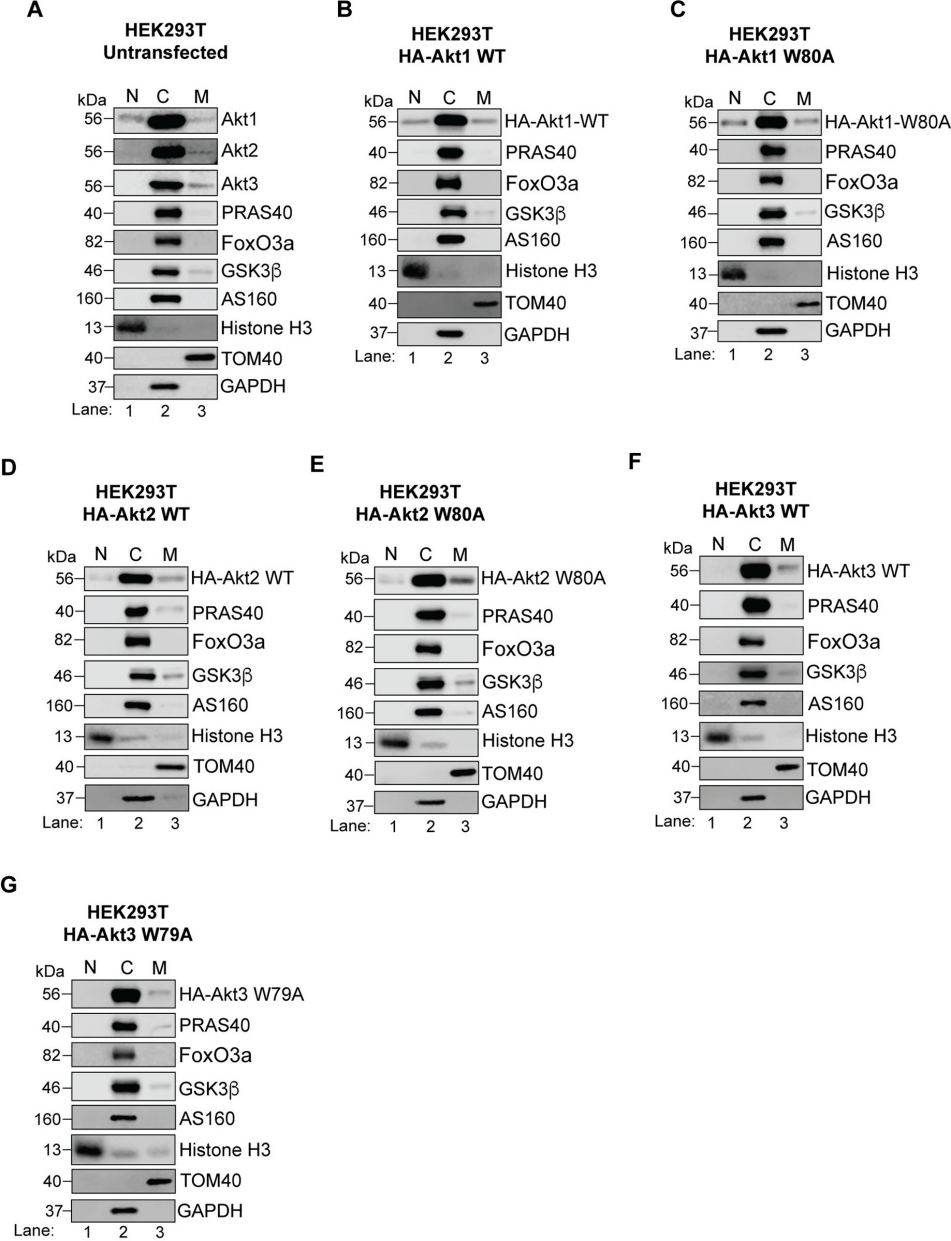
Subcellular fractionation of Akt isoforms in HEK293T cells. HEK293T cells (A) untransfected or (B-G) transfected with the indicated HA-Akt construct were fractionated into nuclear, cytosolic and mitochondrial fractions. The fractions were immunoblotted for endogenous Akt isoforms (A) or for the exogenously expressed HA-Akt isoform (B-G). The fractions were also blotted for the indicated Akt substrates. Histone H3, GAPDH and TOM40 were blotted as the nuclear, cytosolic and mitochondrial controls, respectively. N = Nuclei, C = Cytosol, M = Mitochondria.

Another plausible explanation for the differential substrate preference in HEK293T and HeLa could be the distinct subcellular localization of the expressed Akt isoforms and hence, differential access to the substrates. To test this, we fractionated lysates of untransfected cells and cells expressing the HA-tagged Akt isoforms (WT and W→A) into nuclear, cytosolic and mitochondrial fractions. As shown in Fig 12, the exogenously expressed Akt isoforms mimicked the localization pattern of their endogenous counterparts in HEK293T cells. All three Akt isoforms predominantly localized to the cytosol with a small portion also partitioning into the mitochondrial fraction (Fig 12A–12G). The only apparent difference among the isoforms in HEK293T cells was that while some Akt1 localized to the nuclear fraction as well, there was little to no nuclear localization for Akt2 and Akt3.

**Fig 13:**
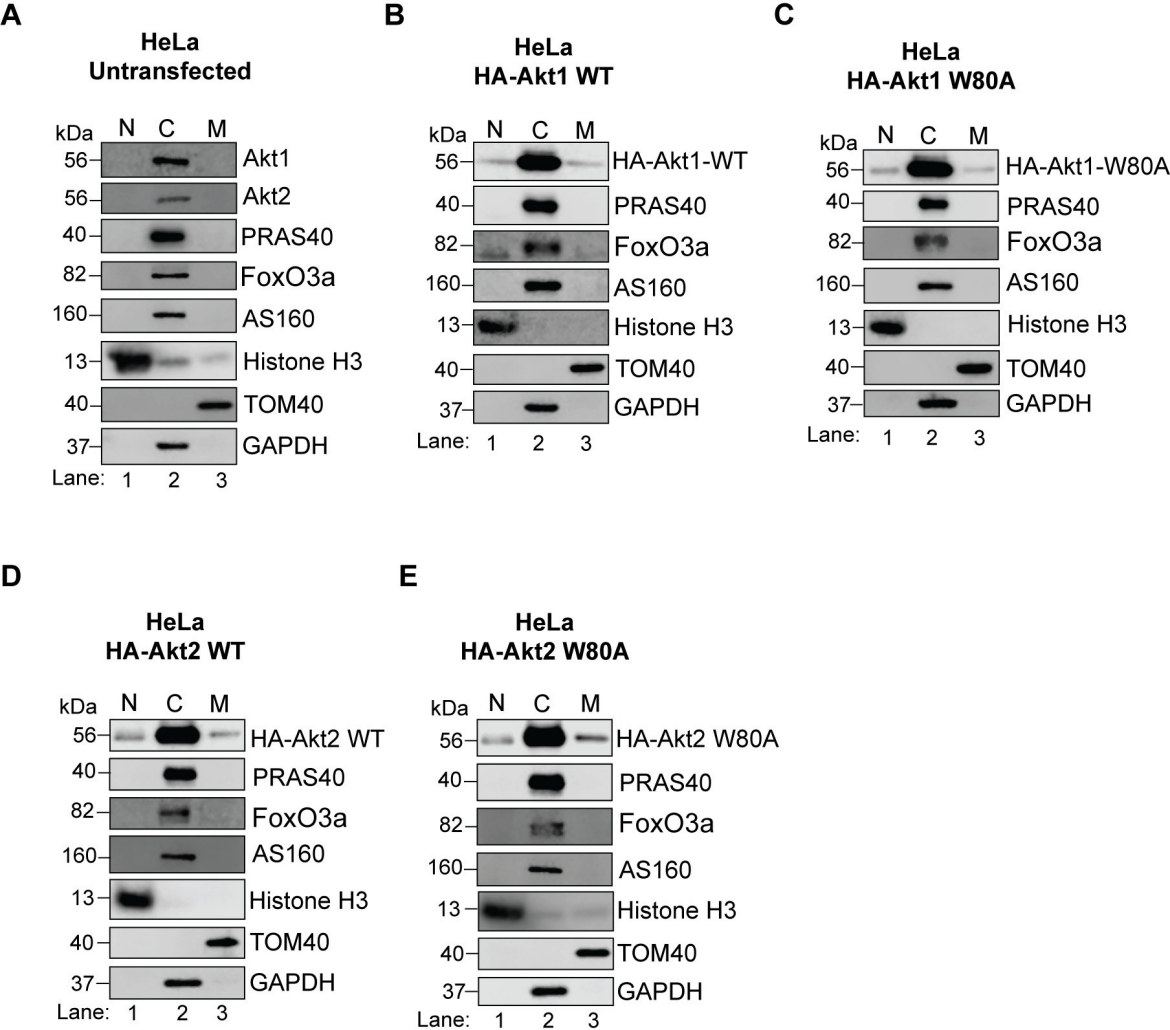
Subcellular fractionation of Akt isoforms in HeLa cells. HeLa cells (A) untransfected or (B-E) transfected with the indicated HA-Akt construct were fractionated into nuclear, cytosolic and mitochondrial fractions. The fractions were immunoblotted for endogenous Akt isoforms (A) or for the exogenously expressed HA-Akt isoform (B-E). The fractions were also blotted for the indicated Akt substrates. Histone H3, GAPDH and TOM40 were blotted as the nuclear, cytosolic and mitochondrial controls, respectively. N = Nuclei, C = Cytosol, M = Mitochondria.

Like HEK293T cells, a major fraction of both isoforms (endogenous and exogenously expressed) localized to the cytosol in HeLa cells (Fig 13). No nuclear or mitochondrial Akt1 or Akt2 was detected at the endogenous level (Fig 13A) whereas the exogenously expressed WT and W80A constructs of both isoforms showed identical pattern with a small portion appearing in both nuclear and mitochondrial fractions (Fig 13B–13E). Based on the comparatively low level of endogenous Akt expression in HeLa cells even in the cytosol (Fig 13A), it is possible that the amount in the other fractions may be below the detection level in untransfected cells. Together, these findings indicate that under steady state conditions, both Akt isoforms fractionate similarly in HeLa cells. These results (Figs 12 and 13) also show that the W79/80A mutants of all isoforms follow the localization patterns of their respective WT proteins, suggesting the W→A mutation does not affect their subcellular localization.

Based on our fractionation experiments, the predominant subcellular location of all Akt isoforms is the cytosol in both cell lines. While the subtle differences observed in the localization of isoforms in HEK293T (Fig 12) *vs* the similar fractionation profile in HeLa cells (Fig 13) could potentially contribute to the observed dominance of Akt1 in HEK293T cells and isoform redundance in HeLa, it does not seem likely considering all the tested Akt substrates in this study also showed a predominantly cytosolic localization in both cell lines (Figs 12 and 13). These results suggest that all expressed Akt isoforms likely had access to the tested substrates. However, despite primarily localizing to the cytosol, the activation status of Akt isoforms may differ. Also, our experiments were conducted under basal conditions which captured a steady state Akt in proliferating cells. It is possible that acute activation of Akt under serum starved, ligand stimulated conditions may lead to a different outcome including changes in subcellular location and/or differential activation of the isoforms. Insulin stimulation of adipocytes resulted in higher recruitment and localization of Akt2 to the plasma membrane as compared to that of Akt1 [38]. However, in another study, the subcellular localization of the three Akt isoforms remained largely unchanged in a serum starved breast cancer cell line stimulated with epidermal growth factor [39].

It is also worth noting that while Akt1 was largely cytosolic, Akt2 and Akt3 in HEK293T and HeLa have been shown to predominantly localize to the mitochondria and nucleus/nuclear membrane, respectively in a previous study [39], and a more recent study has reported majority of Akt2 in the nucleus with some in the cytosol, and Akt3 on the nuclear membrane/envelope in HeLa cells [40]. One explanation for these contrasting results is the method used—both these studies, especially Santi and Lee [39] primarily used immunofluorescence microscopy whereas we used subcellular fractionation/immunoblotting. Since Akt binds to the membranes peripherally through its PH domain, it is possible that the association with the membranes was disrupted during the fractionation procedure. In the nuclear-cytoplasmic fractionation experiments reported in Wainstein *et al* [40], cells were serum starved overnight while our experiments were performed on cells maintained under full serum conditions which may also influence/alter the subcellular localization. In the same study [40], when the authors expressed green fluorescent protein tagged Akt constructs, all three proteins localized to the nuclei as well as the cytoplasm. Both these studies also reported expression of Akt3 in HeLa cells. Notably, Akt3 in HeLa had very low mRNA expression and was undetectable using immunoblotting but could be visualized via immunofluorescence microscopy using a high concentration of the antibody (1:10 dilution) [39]. Wainstein *et al* also reported difficulty detecting expression of Akt3 in HeLa via immunoblotting in whole cell lysates presumably due to inefficient extraction of the nuclear envelope [40]. Therefore, several factors can influence the subcellular localization of Akt isoform, including the growth conditions, expression level, and the methods utilized. Based on our fractionation experiments, the tested Akt substrates mainly localize to the cytosol under basal conditions, suggesting that differential access to substrates is not a major contributor to the differences we observed in isoform dominance in HEK293T *vs* HeLa cells under the conditions tested in this study.

Another difference we noticed in the two cell lines is that the pan-Akt antibody detected two closely migrating but distinct bands in HeLa (Figs 7 and 8). In HEK293T cells, the Akt bands ran almost as one with occasional separation visible as in Fig 2C (lanes 4, 5) upon inhibition of Akt with MK-2206. This likely stems from differential post-translational modification of the isoforms in the two cell lines since all three isoforms in their unmodified forms have very comparable molecular weights (Human Akt1 = 55.7kDa; Human Akt2 = 55.8 kDa; Human Akt3 = 55.8 kDa). This difference is unlikely to be resolved on standard polyacrylamide gel electrophoresis performed under denaturing and reducing conditions on a 10% resolving gel. Also, in our experiments, HEK293T cell lysates consistently showed high basal levels of both pT308 and pS473-Akt, whereas in HeLa cells pT308-Akt detection at endogenous level was challenging. It is possible that phosphorylation of T308 on one or both endogenous Akt isoforms is very tightly and dynamically controlled in HeLa cells; thus, making it technically challenging to detect under steady state conditions. Together, these observations further suggest that Akt isoforms are differentially regulated in different cell lines.

In conclusion, the work presented here used a previously established chemical genetics strategy [29] to determine Akt isoform substrate specificity in two widely used cell lines under basal conditions. So far, the strategy has been used to study function of Akt isoforms during acute insulin receptor signaling where cells are serum starved and stimulated with insulin [29,41–43]. Here, to our knowledge, we used it for the first time to study Akt isoform substrate preference under steady state conditions. This strategy offers a relatively simple and easy method to study Akt isoform preference for a particular substrate in the cell line(s) of interest.

Our work also highlights the inherent cell line differences in Akt isoform dominance, redundance and regulation. It cautions against generalizing the findings and assigning the substrates to a specific isoform of Akt based on data obtained from one cell line. There are many Akt inhibitors currently in clinical trials for various cancers, the majority of which target all three isoforms [44–46]. Since Akt is an important player in regulating several important functions in normal cells too, inhibiting all isoforms is almost certain to cause unwanted side effects. The fact that different isoforms can mediate non-overlapping and, in some cases, even opposing functions, further emphasizes upon the importance of studying and documenting unique roles and/or functional redundancies of Akt isoforms in different cell types/tissues as they relate to normal- and patho-physiology.

## Supporting information

S1 Raw images (PDF)
