# Supplementary material for: Substrate preference of protein kinase B isoforms can vary depending on the cell line
Source: PLoS One. 2024 Mar 19;19(3):e0298322. doi: 10.1371/journal.pone.0298322 (PMC10950239; doi:10.1371/journal.pone.0298322)

Fig 1 - Raw data

Fig 1A

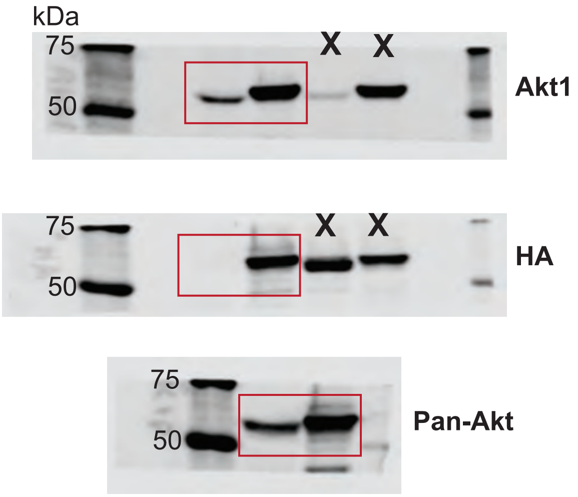

Fig 1B

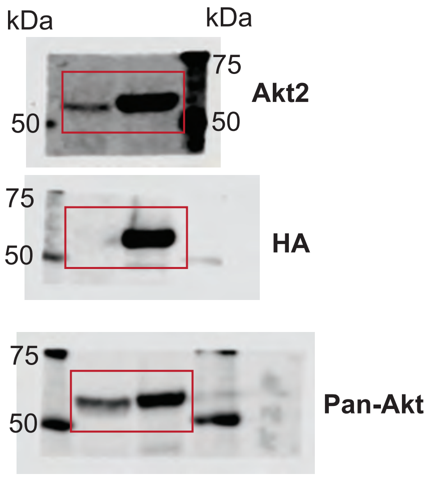

Fig 1C

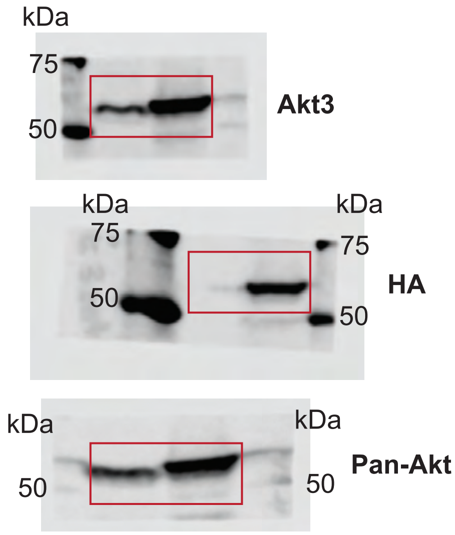

Fig 2 - Raw Data

Fig 2C

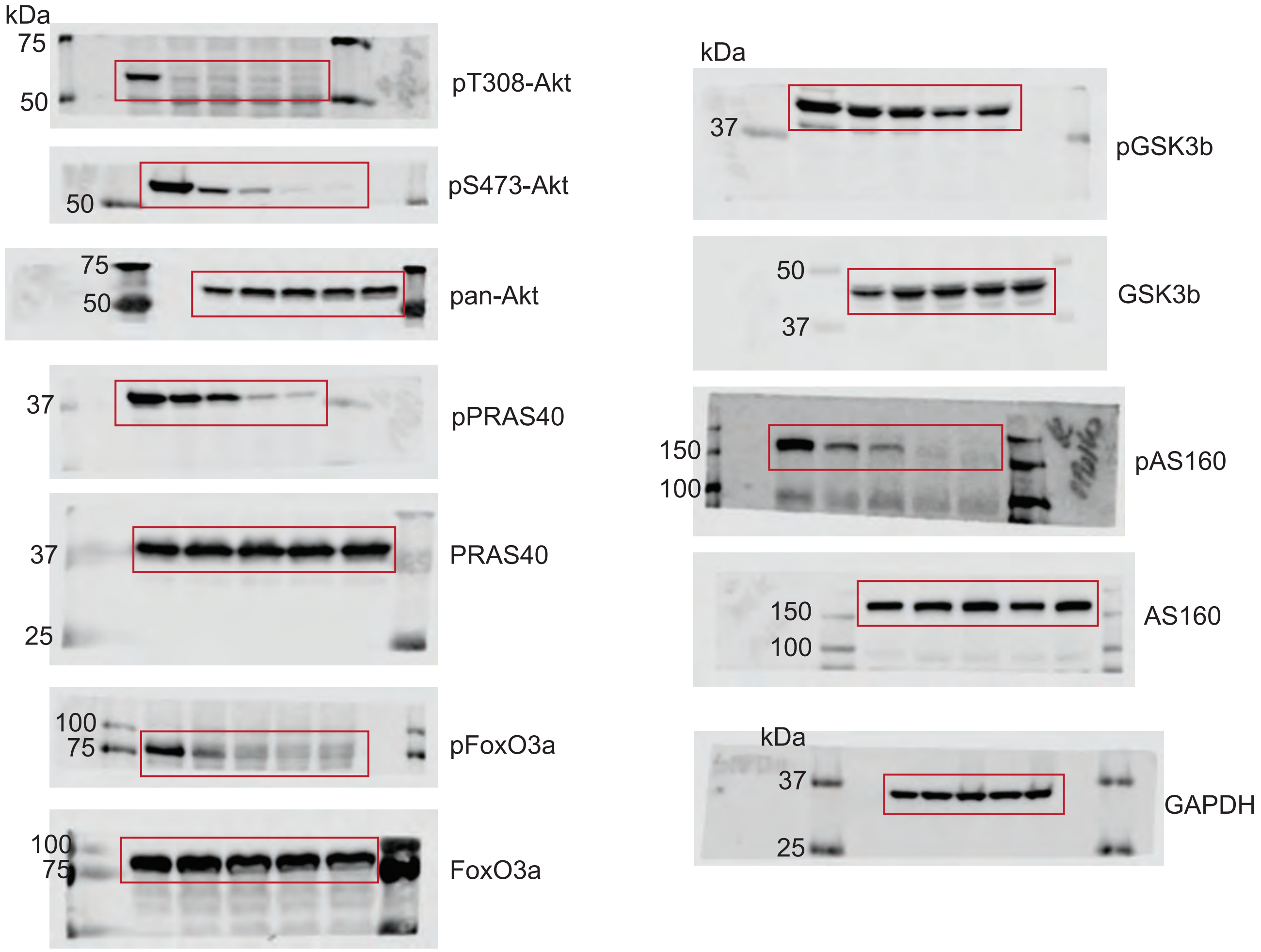

Fig 3 - Raw data

Fig 3A

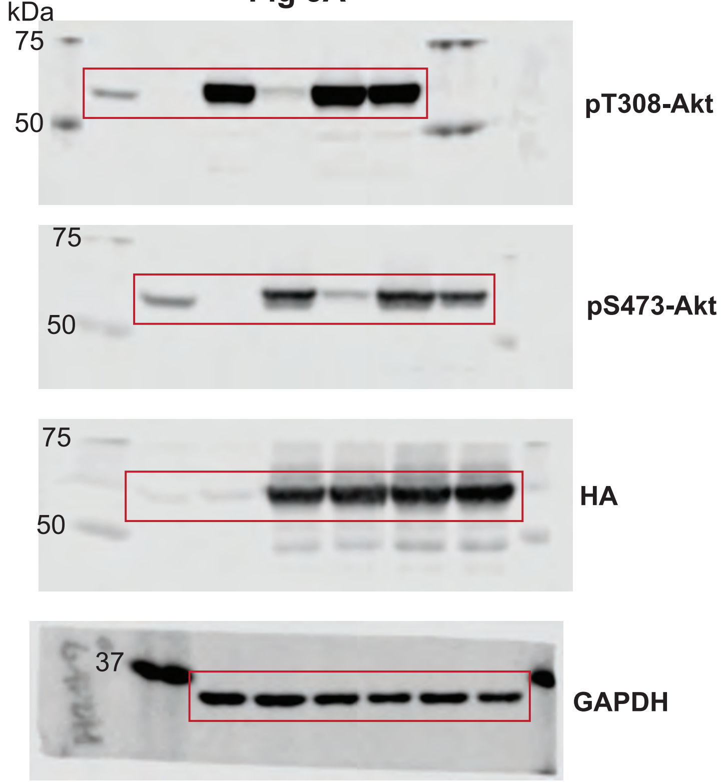

Fig 3B

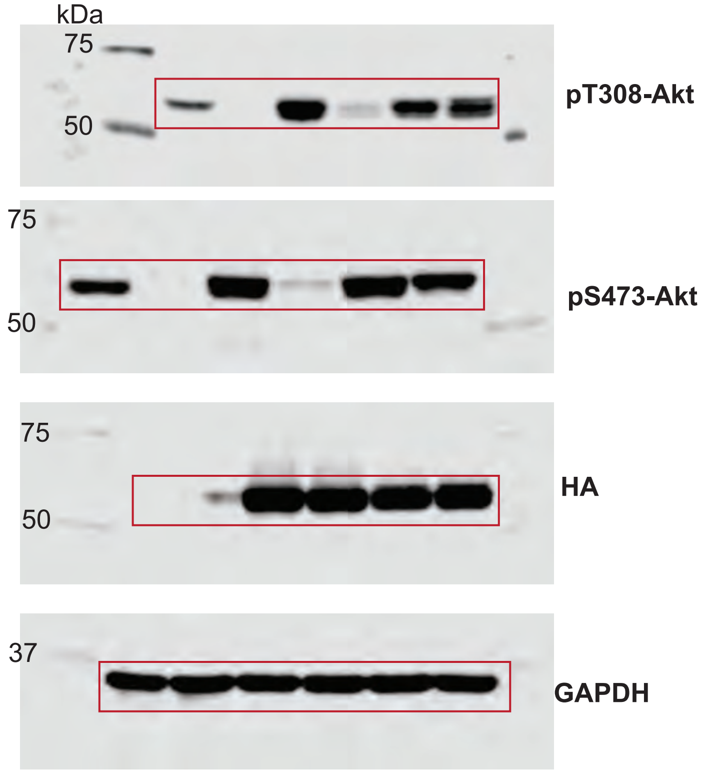

Fig 3C

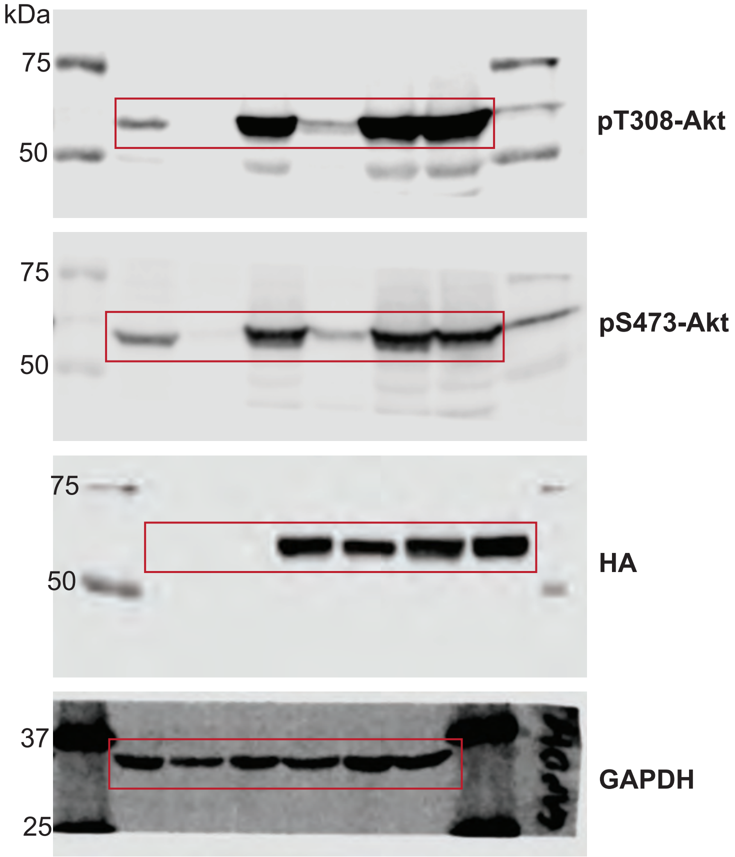

Fig 4 - Raw Data

Fig 4A

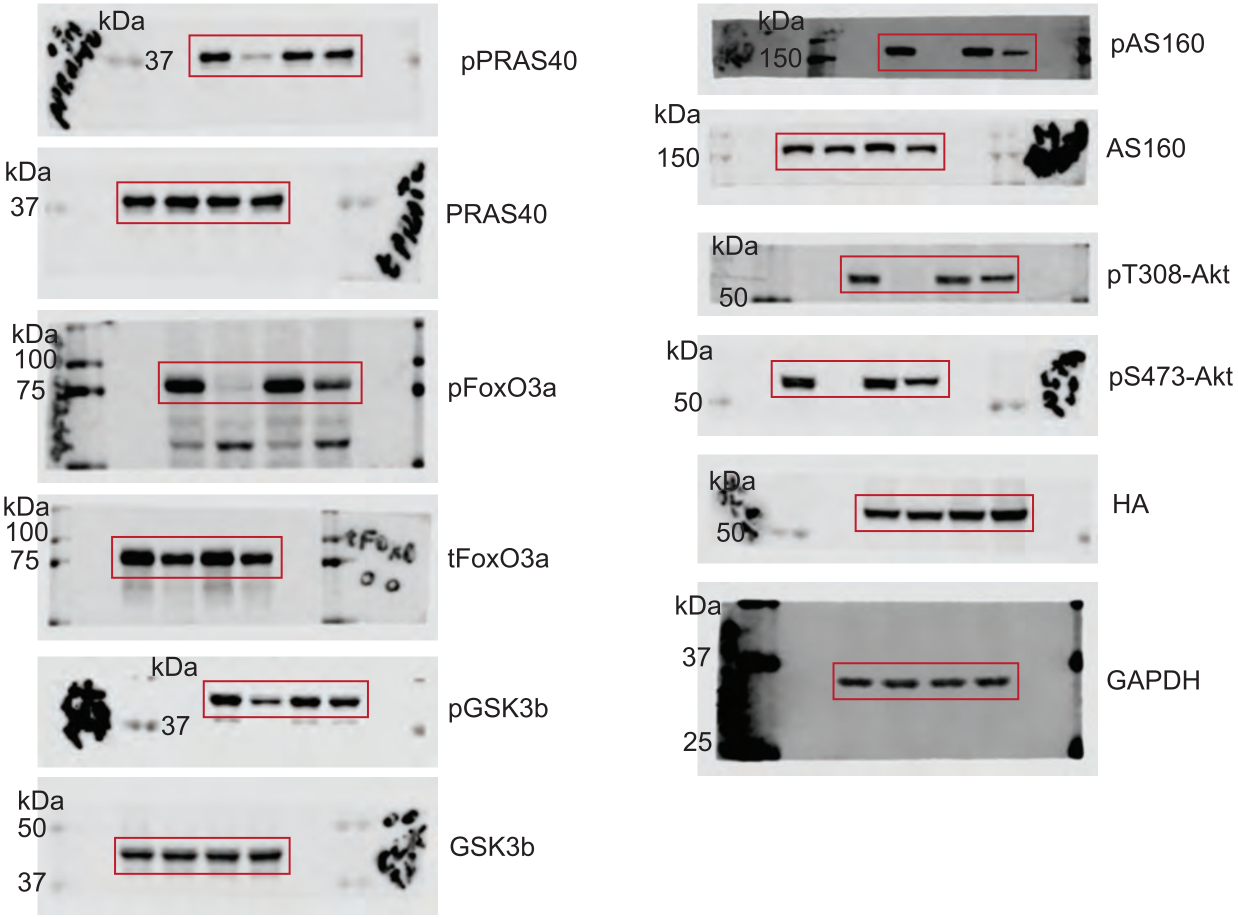

Fig 5 - Raw Data

Fig 5A

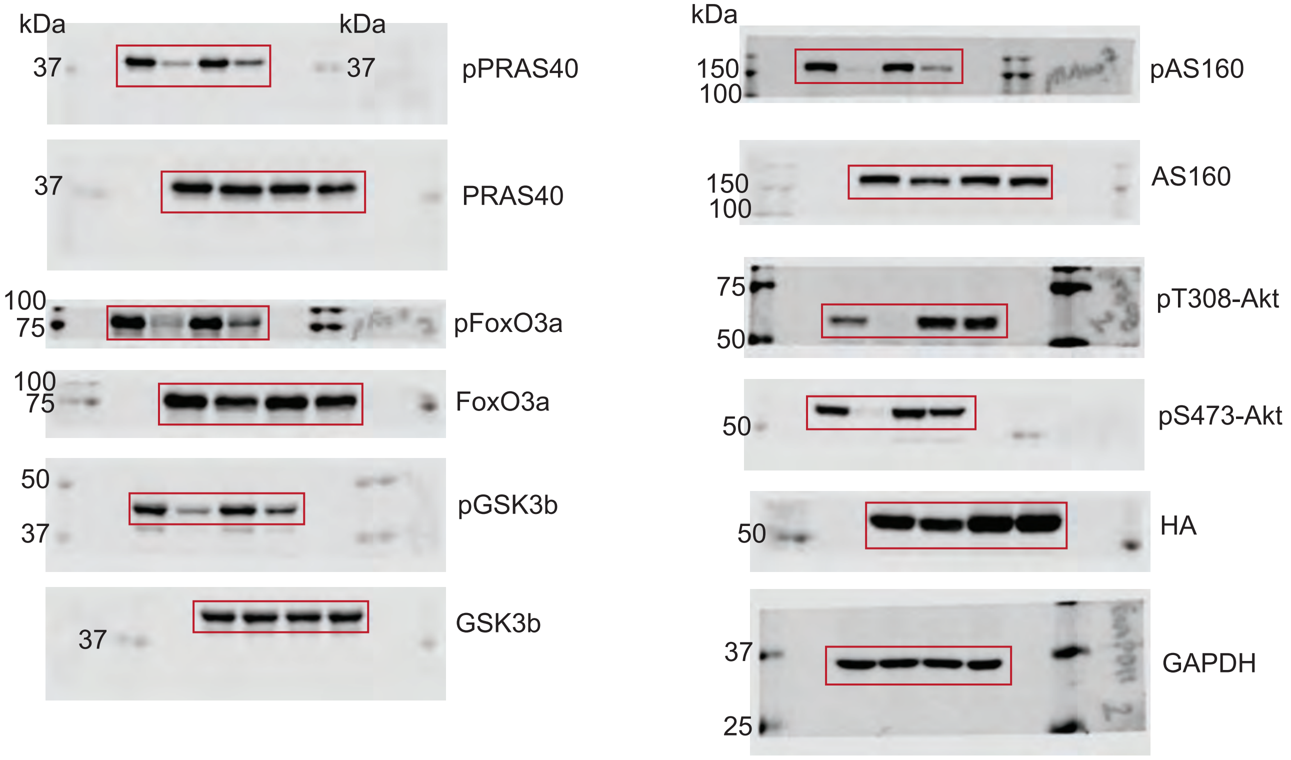

Fig 6 - Raw Data

Fig 6A

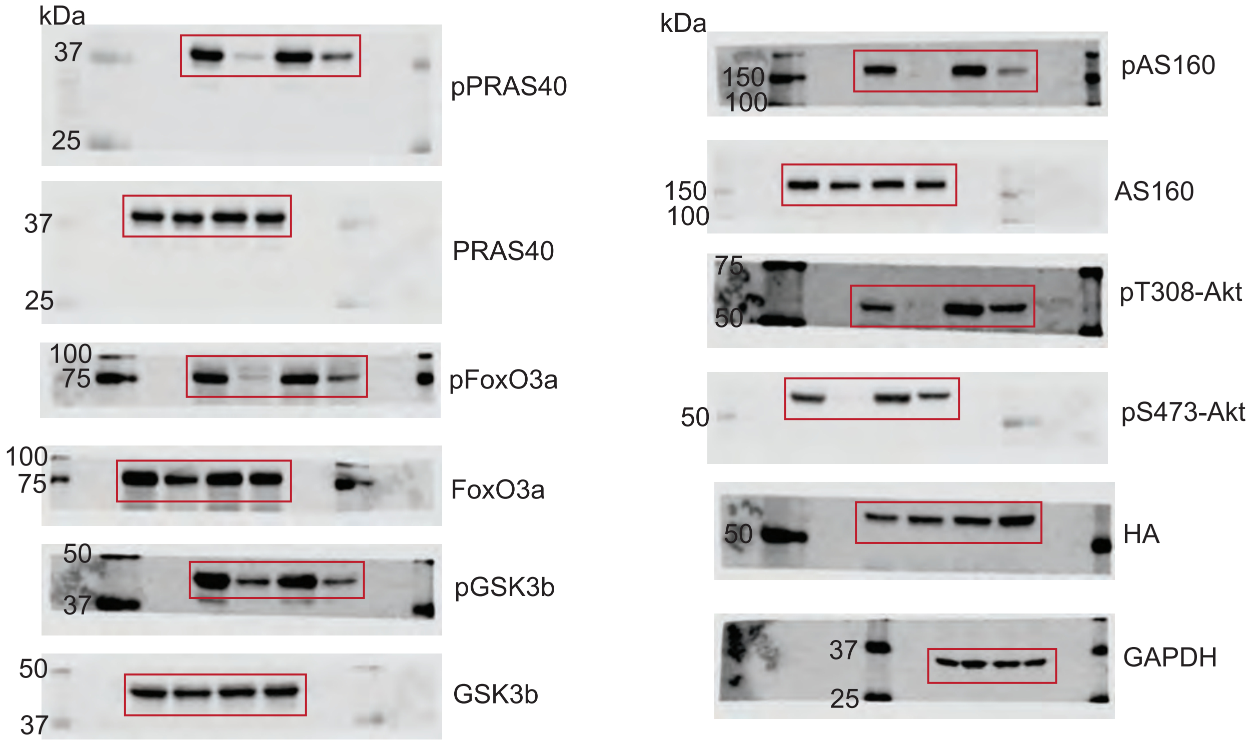

Fig 4B HEK293T HA-Akt1

	pPRAS40		pFoxO3a		pGSK3b		pAS160	
	WT	W80A	WT	W80A	WT	W80A	WT	pAS160 W80A
n1	15.0343758	64.0122792	20.53125	129.628363	28.3956044	80.7841087	1.52682927	94.0587867
n2	13.0327751	31.794116	11.1571977	48.5714286	33.6921006	51.6733467	0.5012456	33.6385973
n3	10.9414977	90.1977722	10.9414977	70.1316016	31.7948718	91.9816495	4.95683654	30.5280528
n4	11.5205655	80.180865	19.1003253	90.5342179	28.3605284	64.196472	5.52070263	100.702576

Fig 5B HEK293T HA-Akt2

	pPRAS40		pFoxO3a		pGSK3b		pAS160	
	WT	W80A	WT	W80A	WT	W80A	WT	pAS160 W80A
n1	26.6220319	26.0270454	24.6266234	42.6291576	43.1351351	87.3170205	14.3078512	75.7733047
n2	12.7588292	30.1970485	15.6810662	17.5361057	25.440806	37.391662	6.34075307	17.0736397
n3	12.234252	19.0823121	16.8762749	31.3676471	14.0905455	13.8221715	6.14517025	43.3901495
n4	14.6561488	13.682048	20.4699663	24.77422	38.0237535	40.1934985	5.29997326	22.5023662
n5	22.0762683	50.7621737	20.0441038	35.0692434	32.6318207	47.9830897	2.6003214	5.26674501
n6	21.0753804	22.0214568	17.755102	52.9539007	15.1571165	36.6449687	0.70588235	14.1309373

Fig 6B HEK293T HA-Akt3

	pPRAS40		pFoxO3a		pGSK3b		pAS160	
	WT	W79A	WT	W79A	WT	W79A	WT	pAS160 W79A
n1	13.0905265	33.1996298	9.45393206	27.6765083	27.5289187	37.2330664	4.77701958	14.4675118
n2	8.09135357	39.6370321	9.46492793	37.1578712	12.9830734	55.4267584	2.30145245	12.5091505
n3	9.12778987	35.829284	25.8231844	33.1458359	16.3601381	17.4635417	3.80175792	20.5941191
n4	18.360689	20.7281451	13.8445639	22.8488746	28.8006112	45.6790634	1.2452371	15.239854

Fig 7 - Raw data

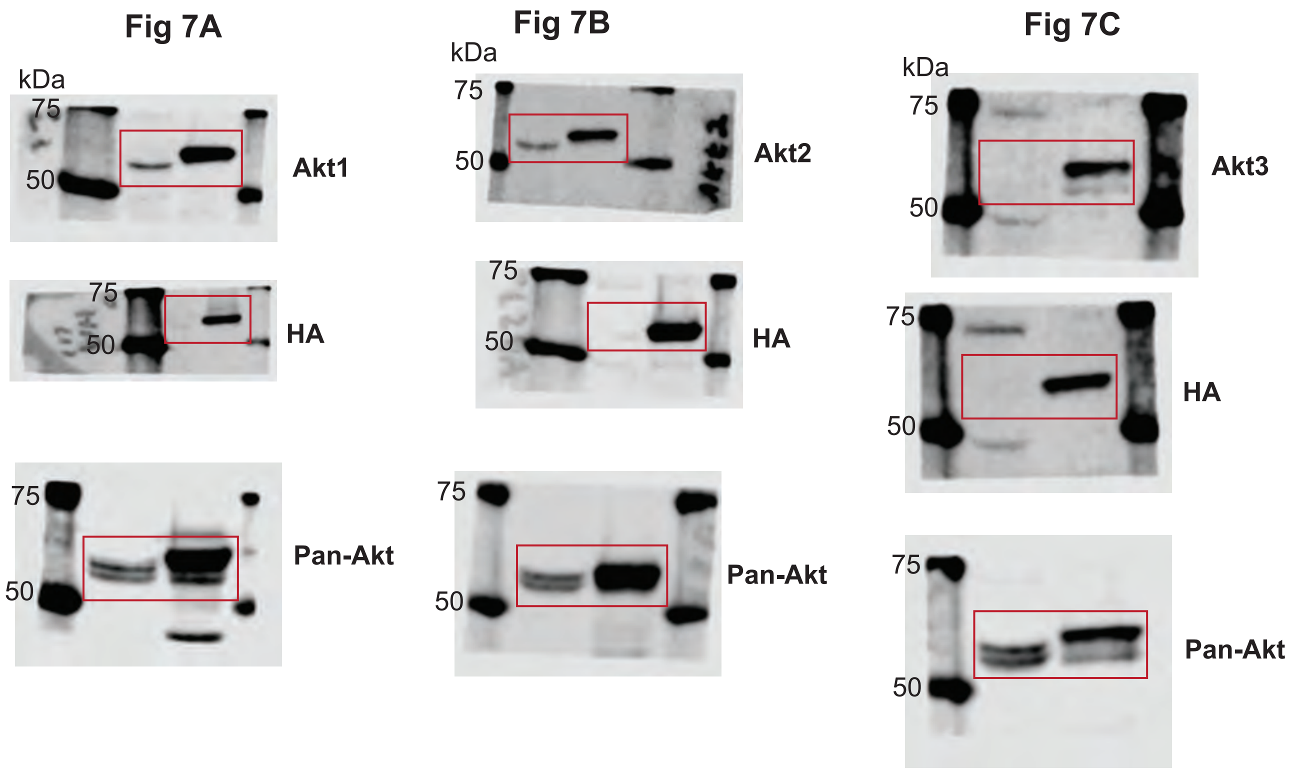

Fig 8A - Raw Data

Fig 8A

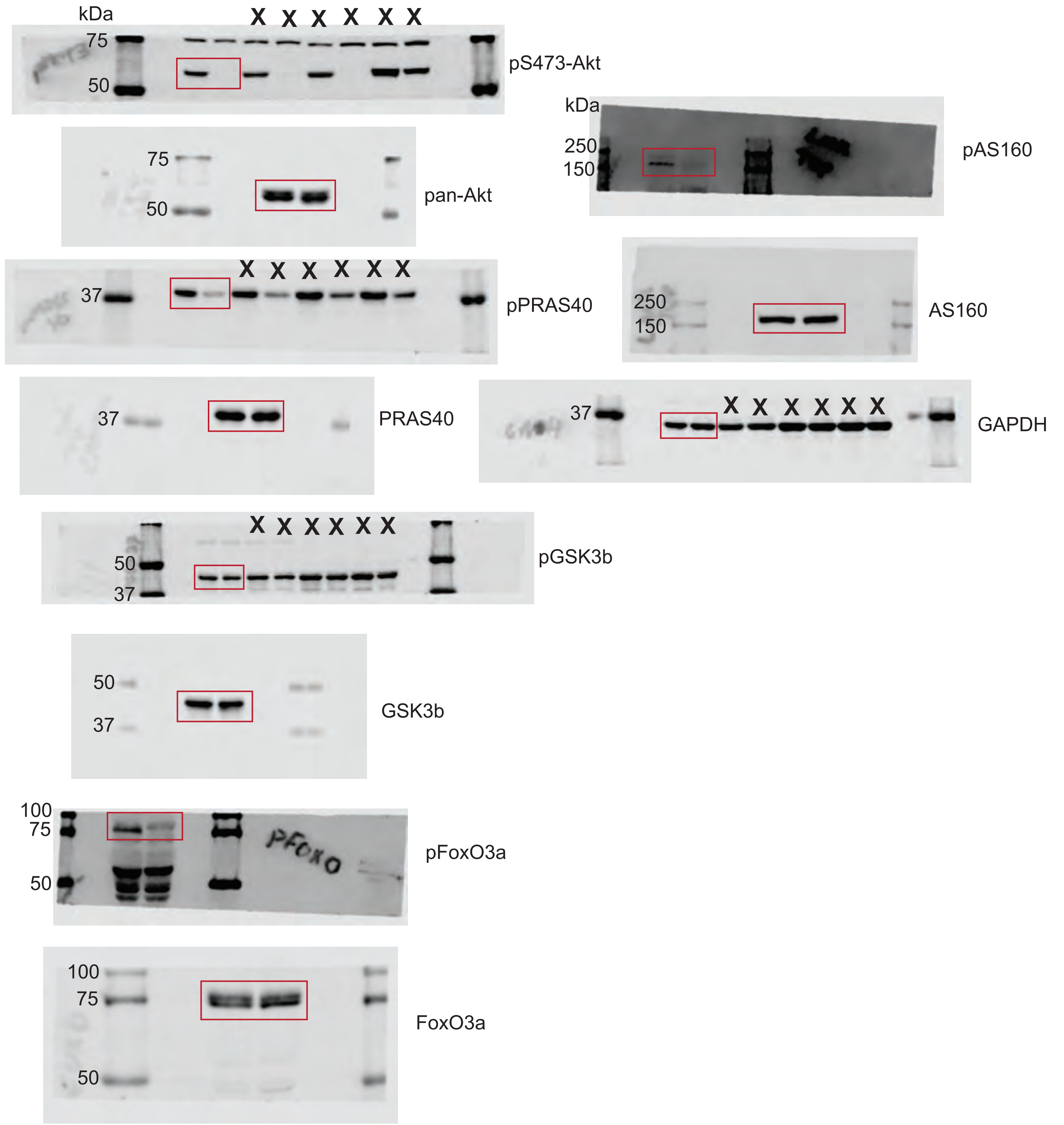

Fig 8B and C - Raw Data

Fig 8B

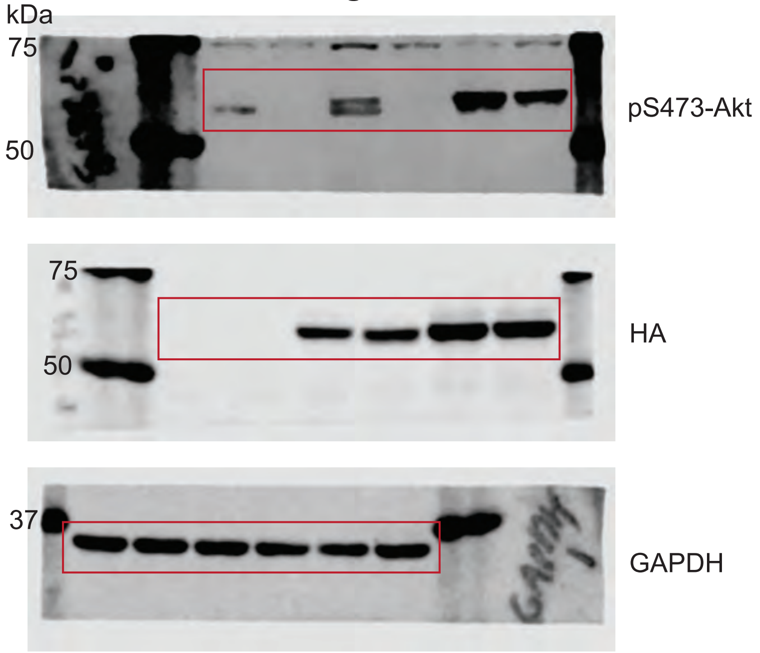

Fig 8C

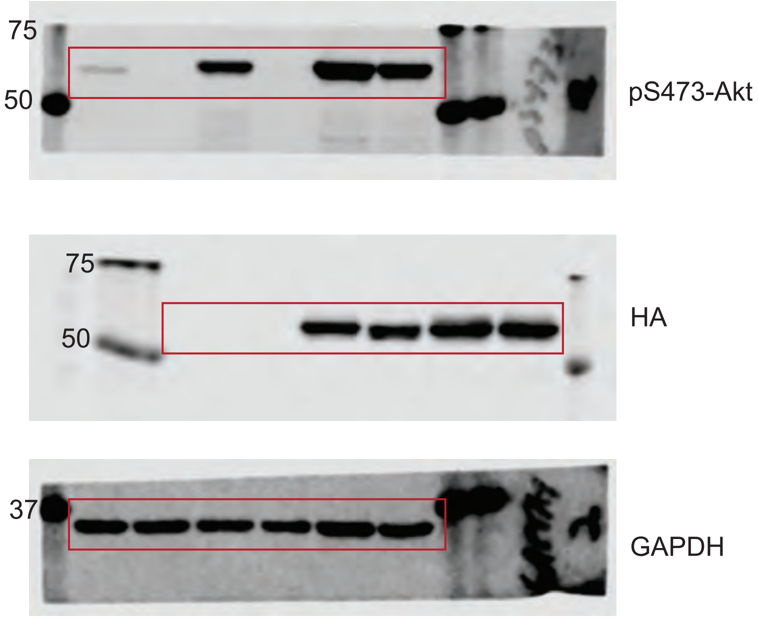

Fig 9 - Raw Data

Fig 9A

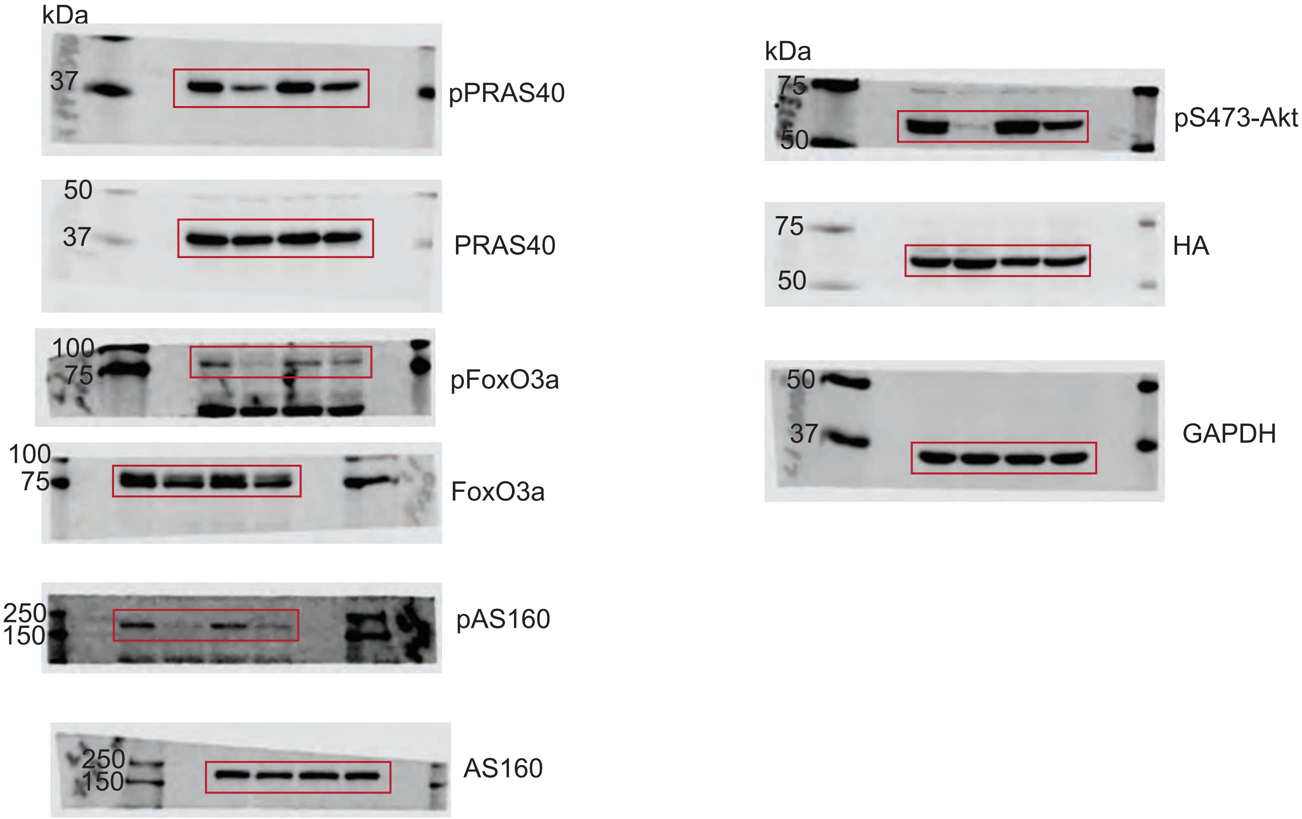

Fig 10 - Raw Data

Fig 10A

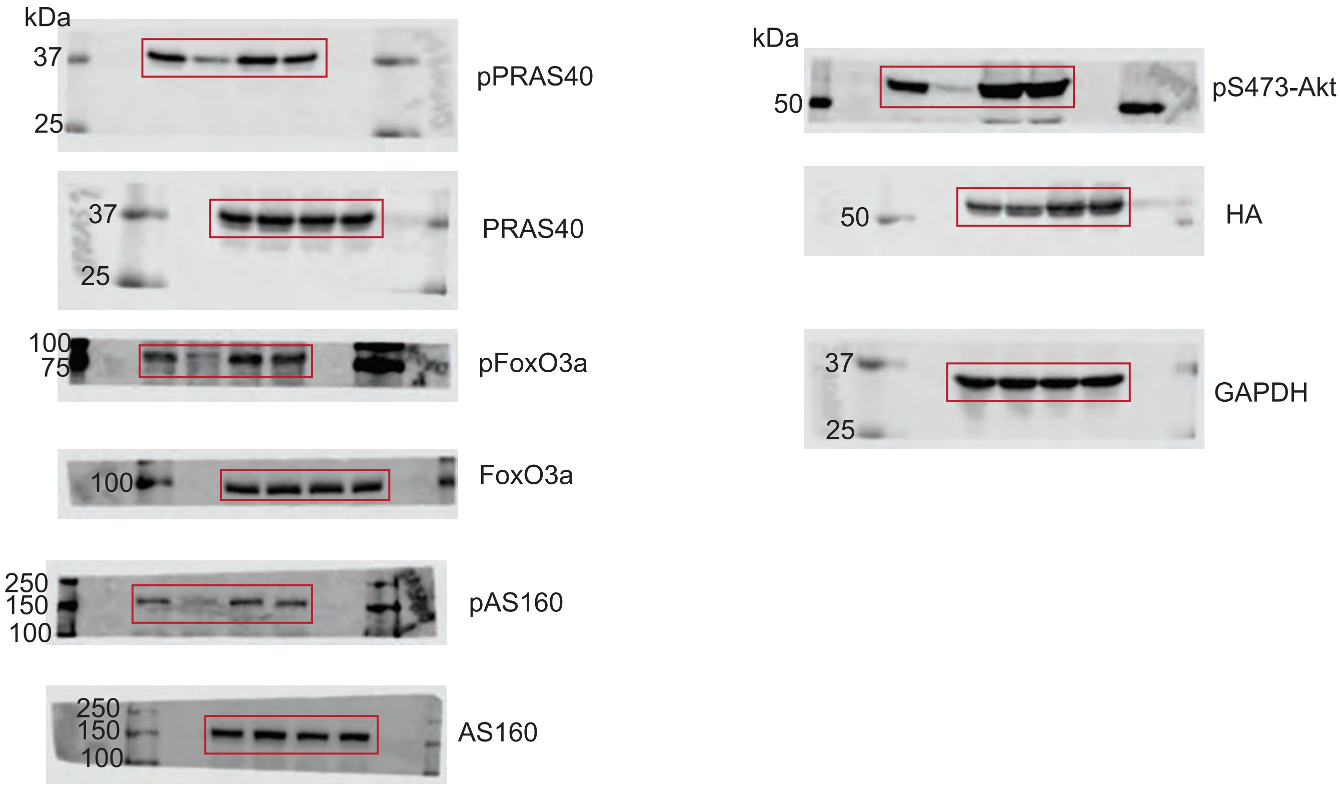

Fig 9B HeLa
HA-Akt1

	pPRAS40		pFoxO3a		pAS160	
	WT	W80A	WT	W80A	WT	W80A
n1	42.4473748	53.1009524	46.2643678	92.3361622	2.88571429	35.2057829
n2	23.1269746	48.4526535	6.38571986	81.0516078	26.2859028	60.9810334
n3	20.9149485	59.939093	46.3308458	97.8813559	12.9966208	56.0515408
n4	28.4131206	71.6138564	37.810534	72.1209345	18.4838396	60.6151306

Fig 10B HeLa
HA-Akt2

	pPRAS40		pFoxO3a		pAS160	
	WT	W80A	WT	W80A	WT	W80A
n1	23.8523163	56.8731642	32.6080056	59.699846	3.4903698	35.5015291
n2	32.4339569	92.5382947	28.5026832	90.1181912	27.794963	75.6659836
n3	35.8769867	78.0293533	46.6650086	118.587805	26.7882071	80.8958418
n4	31.7210866	79.8136041	40.9252325	91.4686139	17.3578466	65.0211182

Fig 11A - Raw Data

Fig 11A (Left panel)

HEK293T
HA-Akt1 W80A

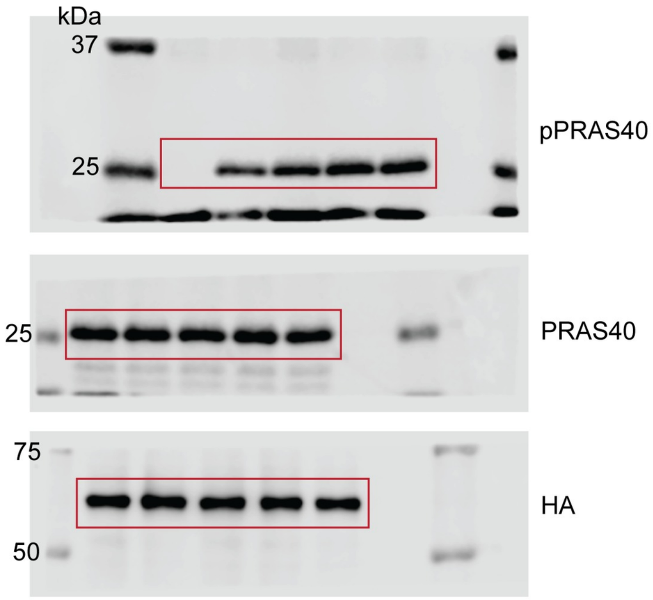

Fig 11A (Middle panel)

HEK293T
HA-Akt2 W80A

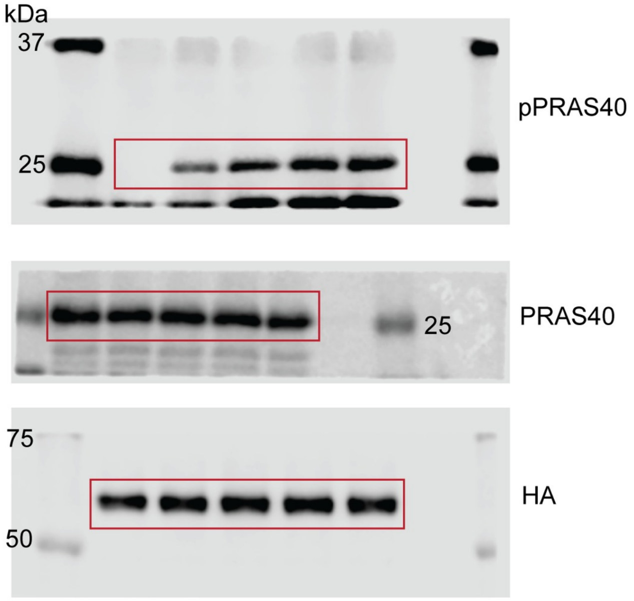

Fig 11A (Right panel)

HEK293T
HA-Akt3 W79A

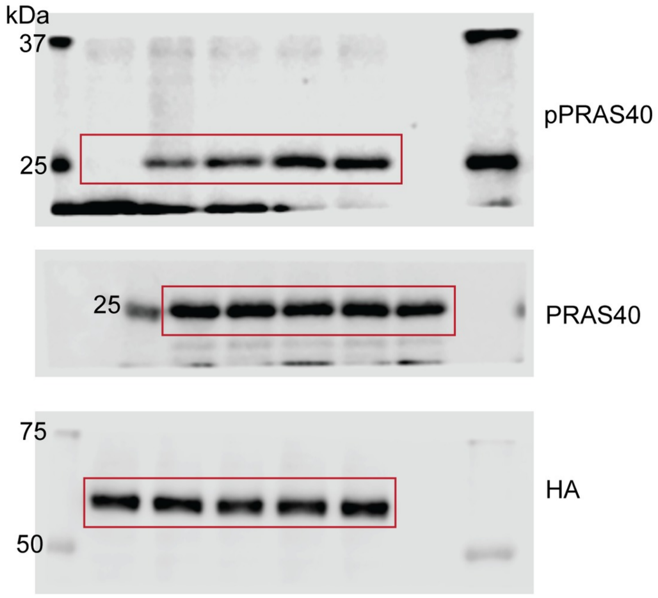

Fig 11B - Raw Data

Fig 11B (Left panel)

HeLa
HA-Akt1 W80A

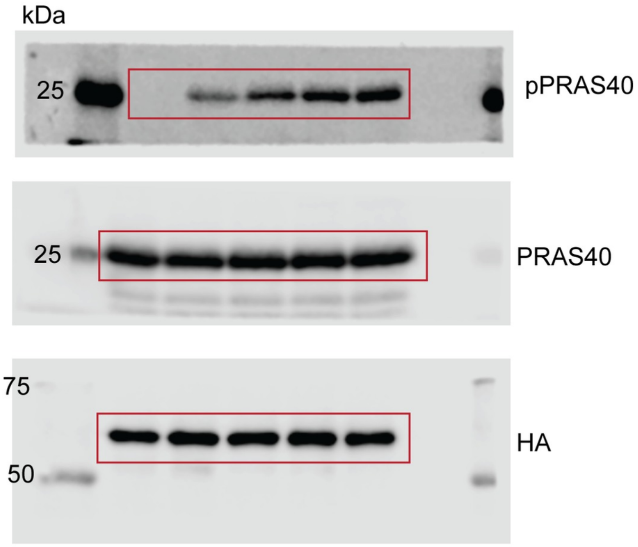

Fig 11B (Middle panel)

HeLa
HA-Akt2 W80A

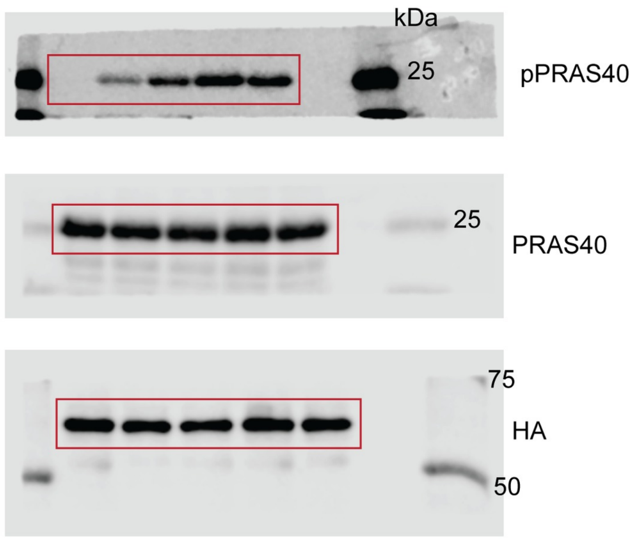

HEK293T
Fig 11C

	Akt1 15 min	Akt2 15 min	Akt3 15 min	Akt1 30 min	Akt2 30 min	Akt3 30 min	Akt1 60 min	Akt2 60 min	Akt3 60 min
**n1**	0.58728412	0.535929	0.50059015	0.98502802	0.85696254	0.72554479	1.13375156	0.83079324	0.87925204
**n2**	0.63081239	0.46233766	0.38632174	0.82292902	0.85953186	0.6276105	0.9480702	0.91069297	0.86200066
**n3**	0.65223181	0.48629522	0.42725173	0.7528176	0.71903192	0.8264089	0.90595459	0.96083969	0.93364502

HeLa
Fig 11D

	Akt1 15 min	Akt2 15 min	Akt1 30 min	Akt2 30 min	Akt1 60 min	Akt2 60 min
**n1**	0.49032677	0.37028694	0.70278762	0.60843169	0.89858178	1.17171169
**n2**	0.35322873	0.41883124	0.55856966	0.74174557	0.90773478	0.90064783
**n3**	0.42845289	0.453215	0.65843621	0.57409734	0.92455418	0.88557181

Fig 12A-B - Raw Data

Fig 12A

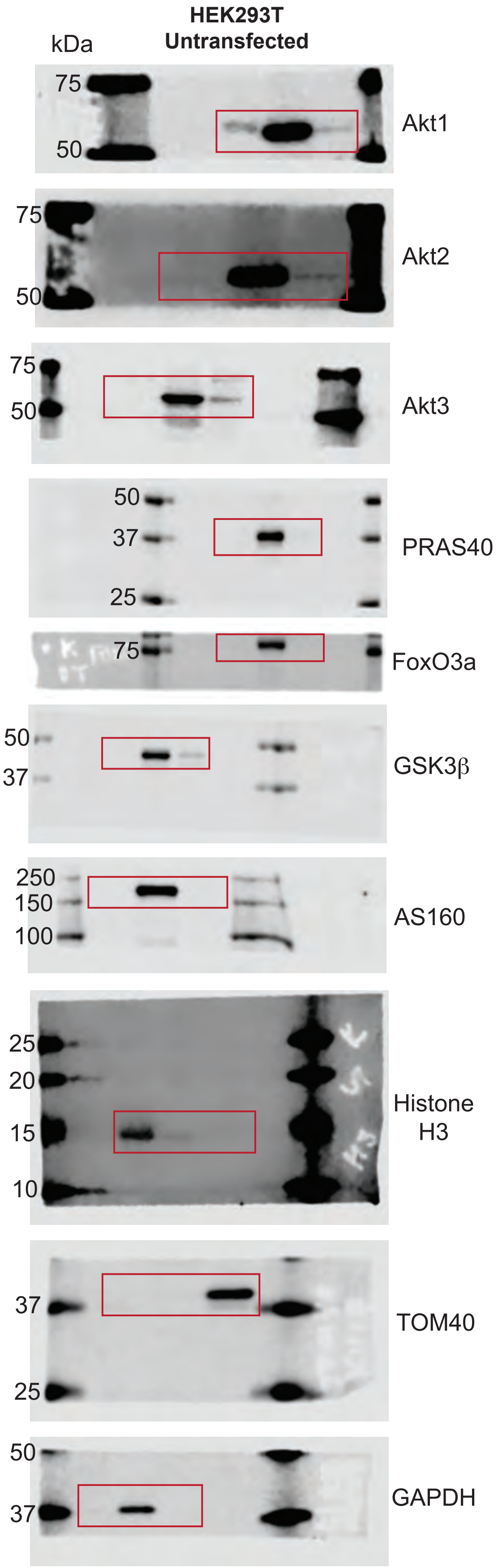

Fig 12B

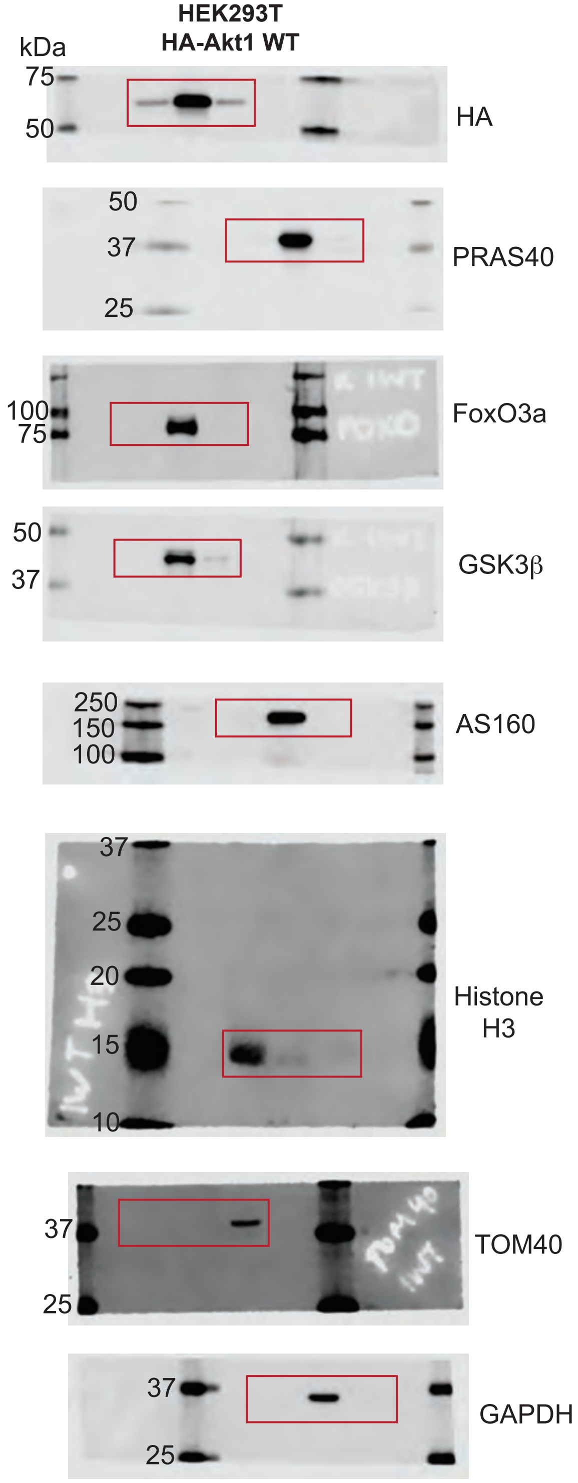

Fig 12C-D - Raw Data

Fig 12C

HEK293T
HA-Akt1 W80A

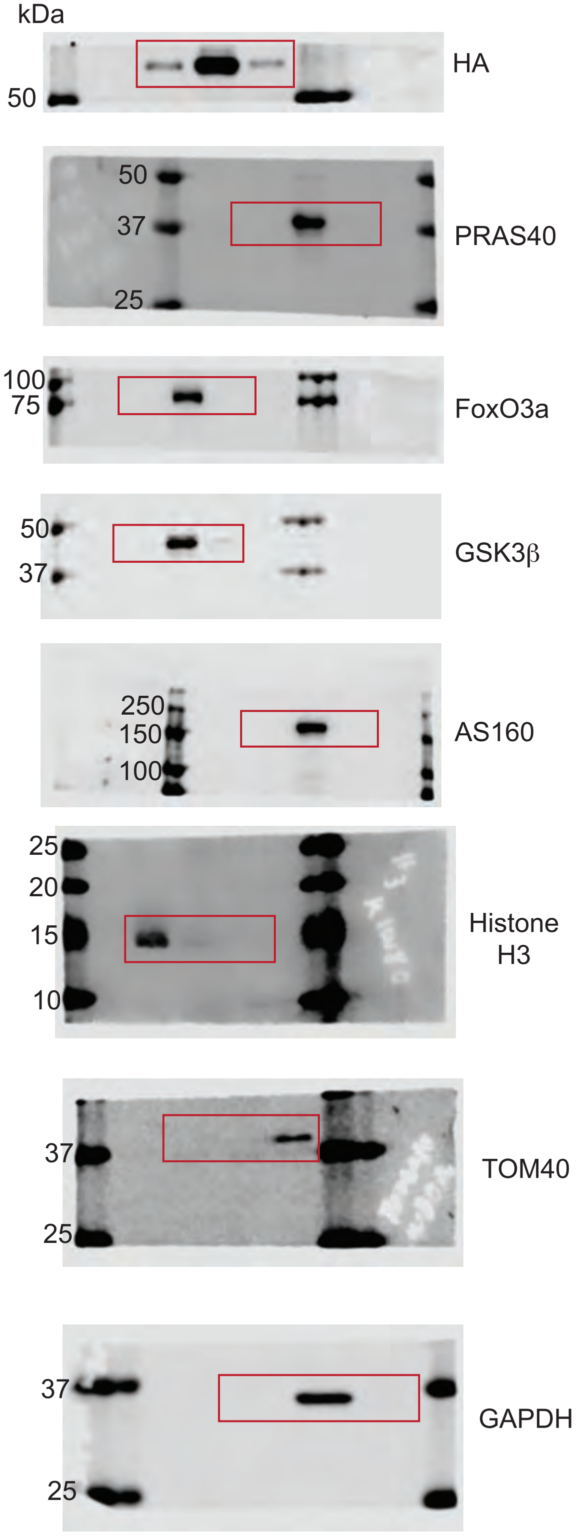

Fig 12D

HEK293T
HA-Akt2 WT

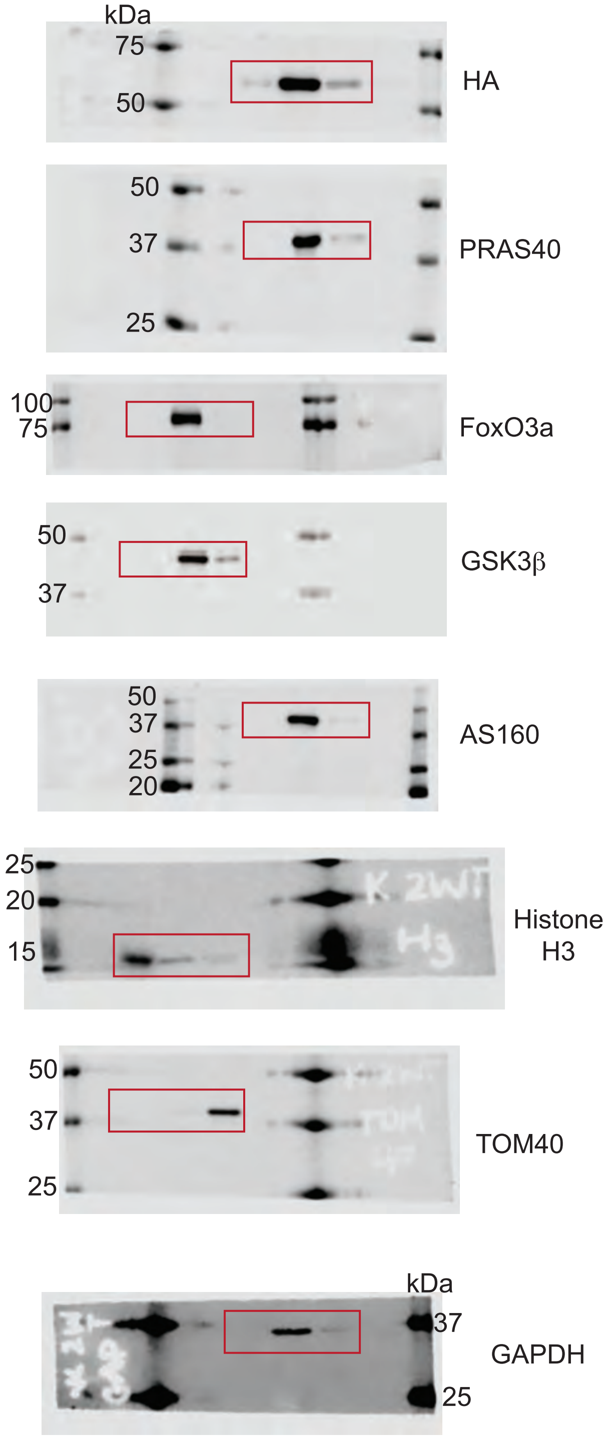

Fig 12E-F - Raw Data

Fig 12E

HEK293T
HA-Akt2 W80A

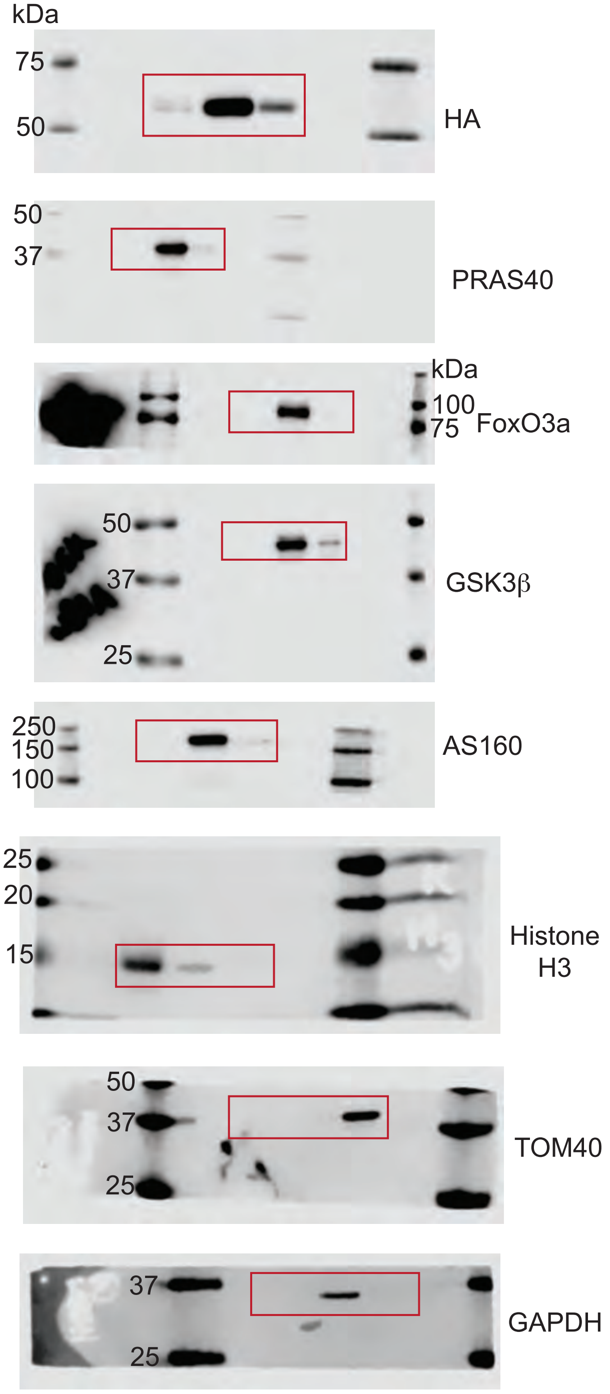

Fig 12F

HEK293T
HA-Akt3 WT

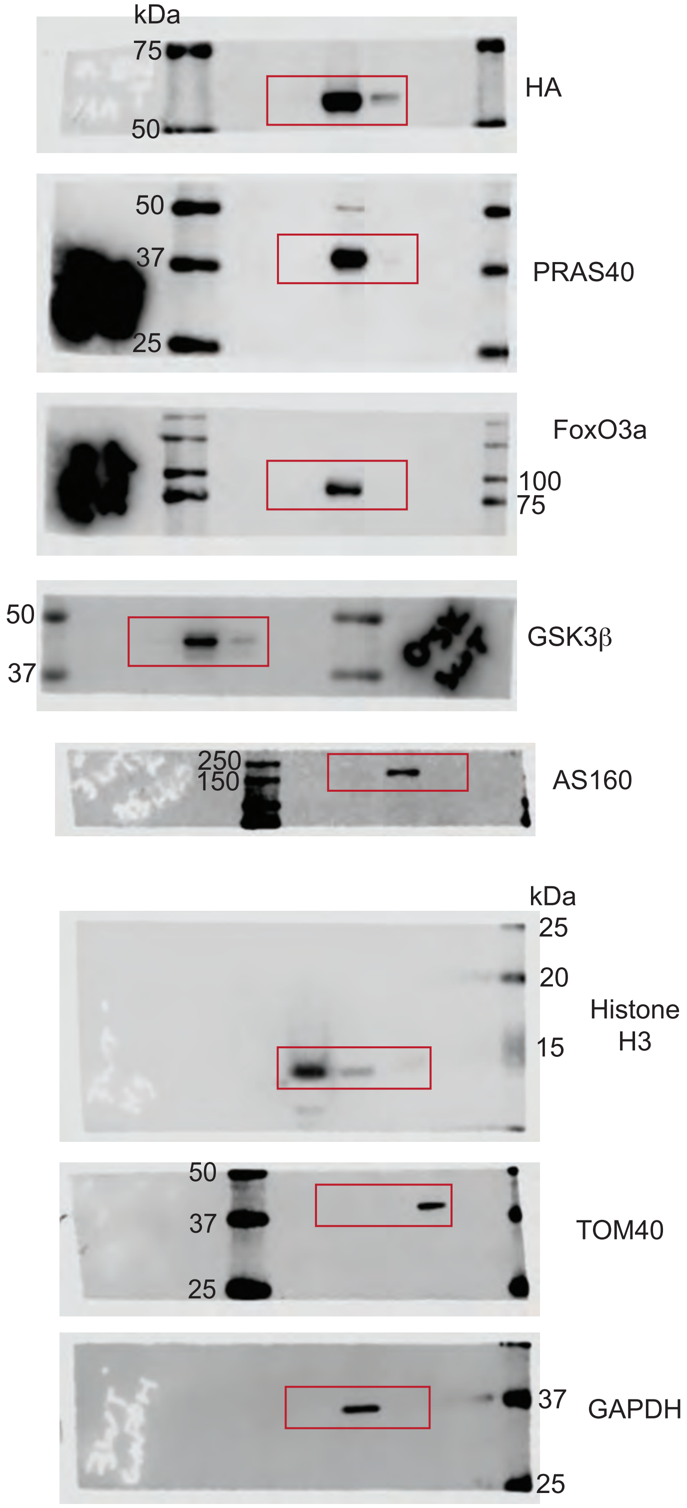

Fig 12G - Raw Data

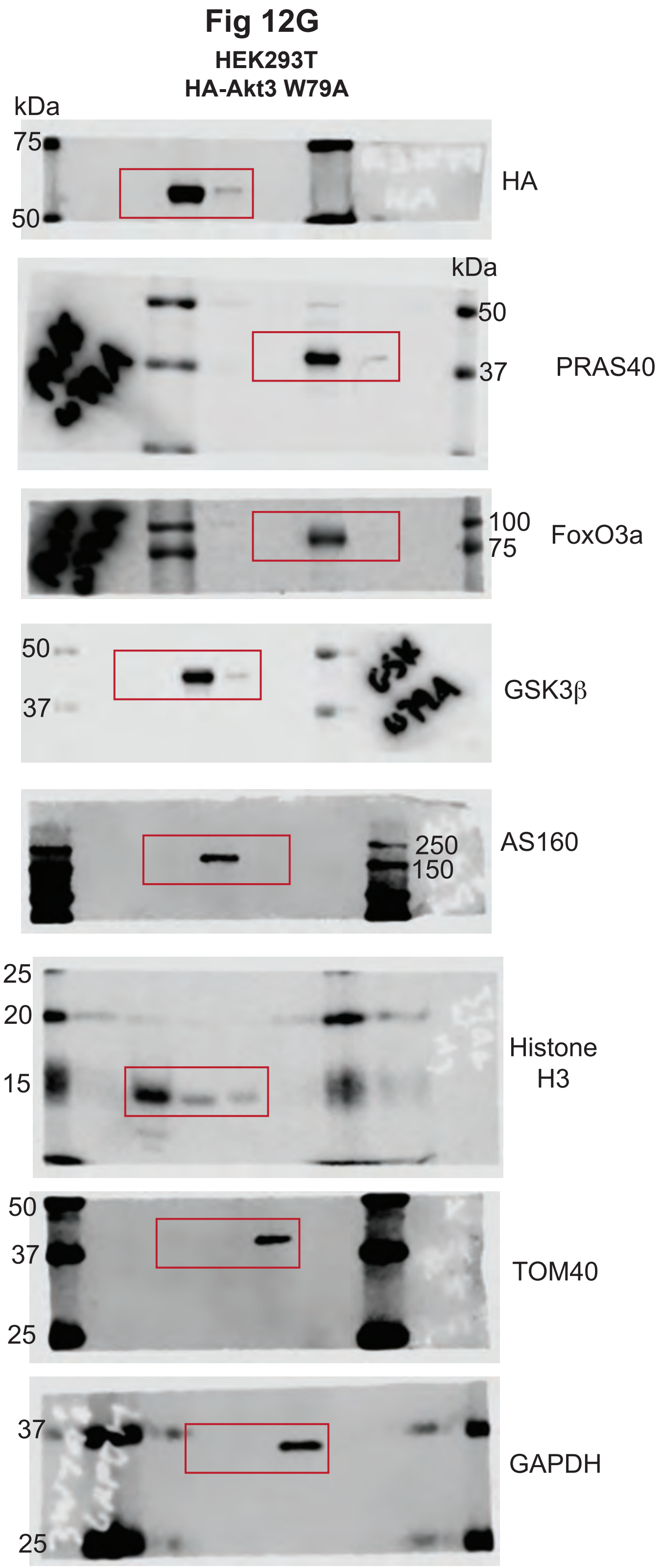

Fig 13A-B - Raw Data

Fig 13A

HeLa
Untransfected

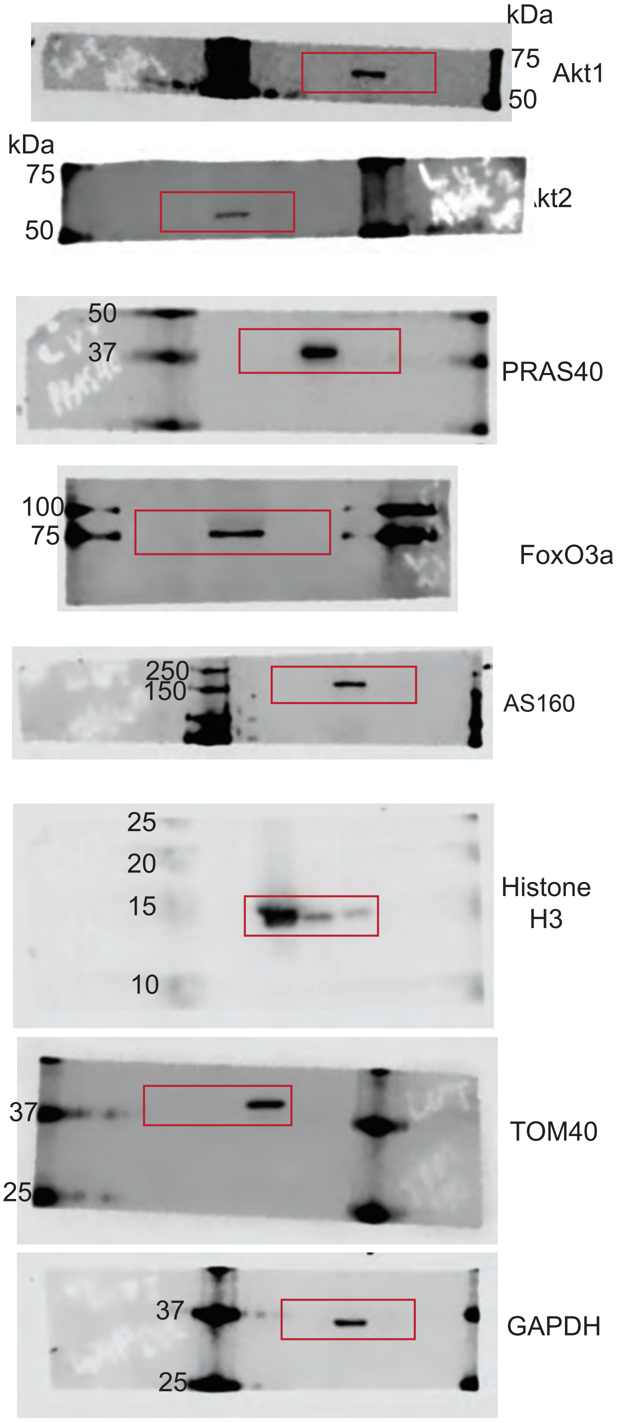

Fig 13B

HeLa
HA-Akt1 WT

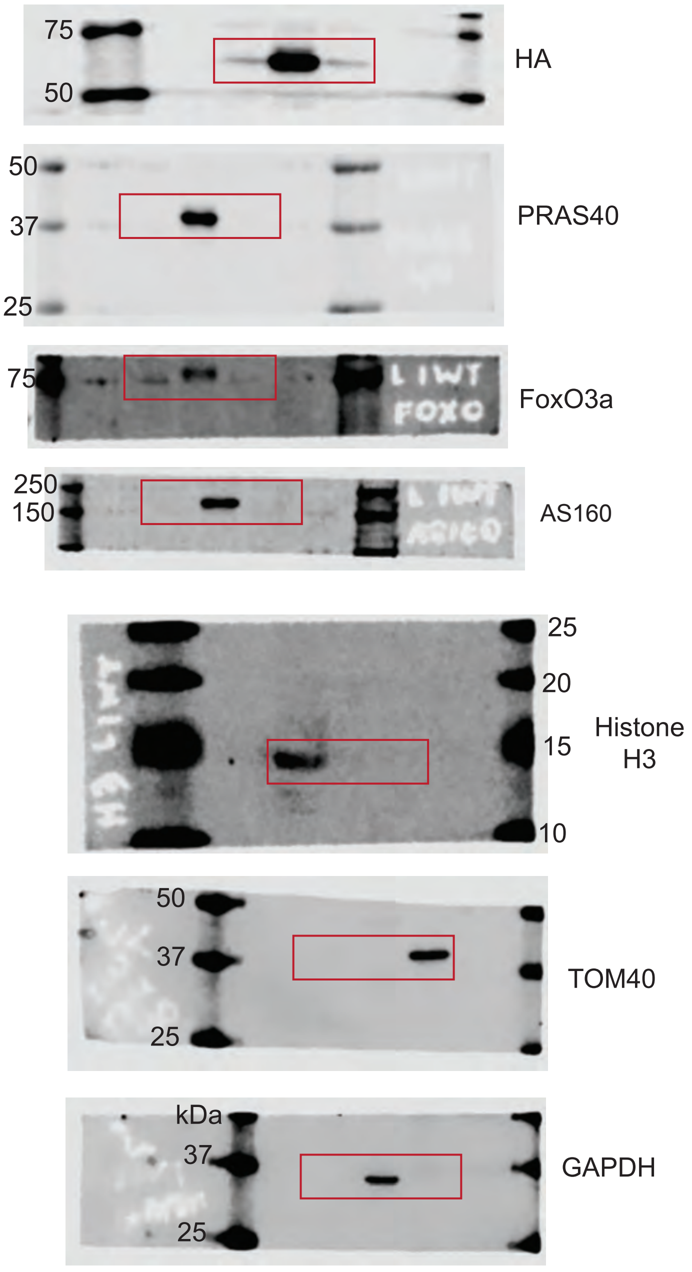

Fig 13C - Raw Data

Fig 13C

HeLa
HA-Akt1 W80A

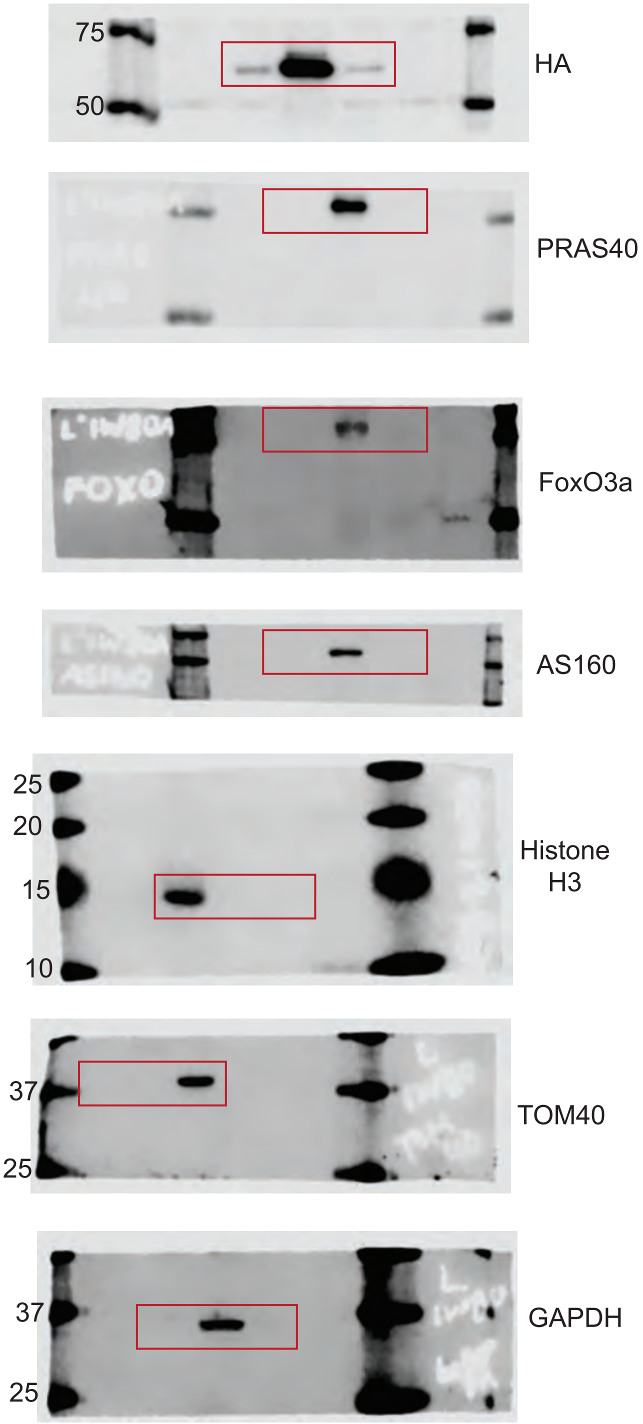

Fig 13D, E - Raw Data

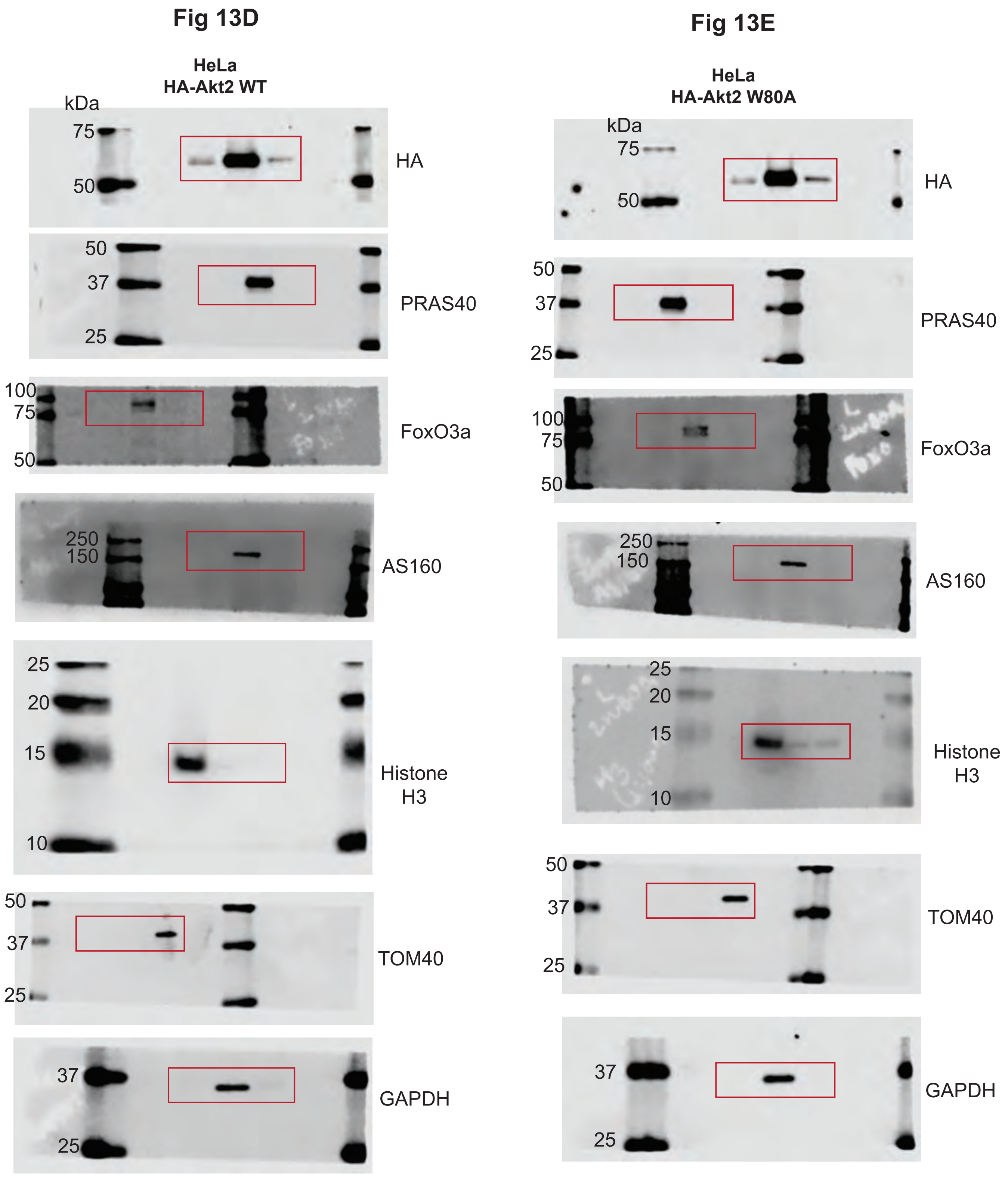

Supplement: S1 Raw images — (PDF) [file pone.0298322.s001.pdf]
